# cPLA2 blockade attenuates S100A7-mediated breast tumorigenicity by inhibiting the immunosuppressive tumor microenvironment

**DOI:** 10.1186/s13046-021-02221-0

**Published:** 2022-02-08

**Authors:** Sanjay Mishra, Manish Charan, Rajni Kant Shukla, Pranay Agarwal, Swati Misri, Ajeet K. Verma, Dinesh K. Ahirwar, Jalal Siddiqui, Kirti Kaul, Neety Sahu, Kunj Vyas, Ayush Arpit Garg, Anum Khan, Wayne O. Miles, Jonathan W. Song, Nidhi Bhutani, Ramesh K. Ganju

**Affiliations:** 1 Department of Pathology, College of Medicine, The Ohio State University, Columbus, OH 43210 USA; 2 Comprehensive Cancer Center, The Ohio State University, Columbus, OH 43210 USA; 3 Department of Microbial, Infection & Immunity, The Ohio State University, Columbus, OH 43210 USA; 4 Department of Orthopaedic Surgery, Stanford University, Stanford, CA 94305 USA; 5 Department of Cancer Biology and Genetics, The Ohio State University, Columbus, OH 43210 USA; 6 Department of Mechanical and Aerospace Engineering, The Ohio State University, Columbus, OH 43210 USA; 7 School of Medicine, Cell Science Imaging Facility, Stanford University, Stanford, CA 94305 USA

**Keywords:** Metastasis, Breast cancer, S100A7, cPLA2, PGE2, Tumor microenvironment

## Abstract

**Background:**

Molecular mechanisms underlying inflammation-associated breast tumor growth are poorly studied. S100A7, a pro-inflammatory molecule has been shown to enhance breast cancer growth and metastasis. However, the S100A7-mediated molecular mechanisms in enhancing tumor growth and metastasis are unclear.

**Methods:**

Human breast cancer tissue and plasma samples were used to analyze the expression of S100A7, cPLA2, and PGE2. S100A7-overexpressing or downregulated human metastatic breast cancer cells were used to evaluate the S100A7-mediated downstream signaling mechanisms. Bi-transgenic mS100a7a15 overexpression, TNBC C3 (1)/Tag transgenic, and humanized patient-derived xenograft mouse models and cPLA2 inhibitor (AACOCF3) were used to investigate the role of S100A7/cPLA2/PGE2 signaling in tumor growth and metastasis. Additionally, CODEX, a highly advanced multiplexed imaging was employed to delineate the effects of S100A7/cPLA2 inhibition on the recruitment of various immune cells.

**Results:**

In this study, we found that S100A7 and cPLA2 are highly expressed and correlate with decreased overall survival in breast cancer patients. Further mechanistic studies revealed that S100A7/RAGE signaling promotes the expression of cPLA2 to mediate its oncogenic effects. Pharmacological inhibition of cPLA2 suppressed S100A7-mediated tumor growth and metastasis in multiple pre-clinical models including transgenic and humanized patient-derived xenograft (PDX) mouse models. The attenuation of cPLA2 signaling reduced S100A7-mediated recruitment of immune-suppressive myeloid cells in the tumor microenvironment (TME). Interestingly, we discovered that the S100A7/cPLA2 axis enhances the immunosuppressive microenvironment by increasing prostaglandin E2 (PGE2). Furthermore, CO-Detection by indEXing (CODEX) imaging-based analyses revealed that cPLA2 inhibition increased the infiltration of activated and proliferating CD4^+^ and CD8^+^ T cells in the TME. In addition, CD163^+^ tumor associated-macrophages were positively associated with S100A7 and cPLA2 expression in malignant breast cancer patients.

**Conclusions:**

Our study provides new mechanistic insights on the cross-talk between S100A7/cPLA2 in enhancing breast tumor growth and metastasis by generating an immunosuppressive TME that inhibits the infiltration of cytotoxic T cells. Furthermore, our studies indicate that S100A7/cPLA2 could be used as novel prognostic marker and cPLA2 inhibitors as promising drugs against S100A7-overexpressing aggressive breast cancer.

**Supplementary Information:**

The online version contains supplementary material available at 10.1186/s13046-021-02221-0.

## Background

Early metastasis to distant organs is a major clinical hurdle in improving the overall and recurrence-free survival of invasive breast cancer patients. Inflammatory immunosuppressive tumor microenvironment (iTME) contributes to cancer cell survival, proliferation, aberrant angiogenesis, early metastasis, and resistance to established chemo or hormonal therapies [1]. S100A7 (Psoriasin) is a pro-inflammatory secreted protein that regulates breast cancer growth and metastasis [2–4]. Previous reports from others and our group have shown that S100A7 significantly contributes to breast tumor growth [2–5]. S100A7 mediates breast cancer growth by enhancing inflammatory signaling cascades [5, 6]. In addition, S100A7 regulates the expression of various pro-inflammatory cytokines such as IL1-α, CXCL-1/8, oncostatin-M, and IL-6 [5, 7]. S100A7 has been shown to mediate its oncogenic effects through its receptor; Receptor for Advanced Glycation End-products (RAGE) [8]. However, the downstream inflammatory oncogenic signaling cascades essential for breast tumor growth and metastasis have not been explored. Therefore, understanding the molecular mechanisms that regulate aggressive tumor growth and metastasis may address an unmet medical necessity for invasive breast cancer.

Sustained inflammation is one of the major hallmarks of breast carcinogenesis [9]. Cytosolic phospholipase A2α (cPLA2) plays an important role in driving inflammation-associated cancer [10–15]. In addition, it contributes to the biosynthesis of inflammatory lipids such as Prostaglandin E2 (PGE2). PGE2 promotes the recruitment of different immune suppressive cells including macrophages in the TME [16]. Macrophages can be classified into two potential subtypes M1 and M2, where M2 exhibits pro-tumor functions [17, 18]. PGE2 was reported to affect the polarization of macrophages in different human malignancies [16, 19]. Recently, a chemical inhibitor against cPLA2 has been proven very effective against aberrant angiogenesis in basal-like invasive breast cancer [20]. S100A7 is highly expressed in psoriasis and cPLA2 inhibitors are considered as potent anti-psoriasis agents [21, 22]. Surprisingly, psoriasis predisposes patients to develop cancer [23–25]. However, the crosstalk between S100A7 and cPLA2 in regulating aggressive breast cancer growth and metastasis and its downstream effects in iTME are not known.

In the present study, we performed an in-depth analysis of S100A7 and cPLA2 in breast cancer progression and metastasis using multiple breast cancer cell lines and patient samples. We further exploited different preclinical mouse models including mS100a7a15-overexpressing bi-transgenic and humanized patient-derived xenograft (Hu-PDX) mouse models to analyze the pharmacological inhibition of cPLA2 in attenuating S100A7-induced pro-tumor effects. Our results reveal that higher co-expression of S100A7 and cPLA2 aggravate breast tumor growth and distant metastasis by enhancing the iTME and correlates with worse recurrence-free survival. Altogether, this study identifies S1007/cPLA2 signaling as a promising target against metastatic breast cancers and demonstrates the efficacy of pharmacological inhibition of cPLA2 to improve the clinical outcome of S100A7-overexpressing breast cancer patients.

## Materials and methods

### Cell culture and other reagents

Human breast carcinoma cell lines MDA-MB-231 and MDA-MB-468 were obtained from ATCC. MVT-1 cells (derived from MMTV-c-Myc; MMTV-VEGF bi-transgenic mice) were obtained from Dr. Johnson. MVT-1 cells were cultured as described [5, 26]. MDA-MB-231 cell lines were transfected with pIRES2-EGFP-hS100A7 or pIRES-2-EGFP using Lipofectamine-2000 reagent as per manufacturer’s instructions and stable S100A7 overexpressing clones were generated using G418 selection (500 mg/mL). Stable S100A7-downregulated MDA-MB-468 cells and PLKO.1-puro vector control cells were cultured as described [2]. BMDM were also isolated from wild-type 6-weeks-old female mice and differentiated into macrophages as described [27]. Dulbecco’s modified eagle medium, fetal bovine serum, penicillin and streptomycin antibiotics, trypsin, and ethylenediaminetetraacetic acid were obtained from Gibco BRL (Grand Island, NY, USA). RAGE Antagonist (FPS-ZM1) and recombinant S100A7 were purchased from Calbiochem and Novus Biologicals respectively.

### Mouse models

The NSG (NOD scid gamma mouse) mice were obtained from the OSU animal core facility. TetO-mS100a7a15 mice were kindly provided by Dr. Yuspa (NIH). TetO-mS100a7a15 mice [21] were cross-bred with MMTV-rtTA mice to generate bi-transgenic MMTV-mS100a7a15 mice. Transgenic littermates were genotyped by PCR. Female MMTV-mS100a7a15 mice were fed with Dox-chow 1 g/kg (Bio-Serv), and mice with a normal diet served as controls. FVB-Tg(C3-1-TAg)cJeg/JegJ mice (Stock No:013591) were purchased from Jackson laboratory. The metastatic triple-negative breast cancer patient-derived xenograft (TM00096) was purchased from Jackson Lab and the humanized-PDX mouse model was developed as described previously [28, 29]. All mice were kept in The OSU’s animal facility in compliance with the guidelines and protocols approved by the OSU-IACUC. MVT-1 cells (1X10^5^) were injected into the mammary glands of mS100a7a15 overexpression bi-transgenic mice. Transgenic mice injected with MVT-1 cells were fed either with Dox-chow 1 g/kg (Tet-on) or a normal diet (Tet-off). Tumors were measured weekly with external calipers and volume was calculated as described earlier and animals were sacrificed and tumors were excised [5].

### Flow cytometry

Freshly prepared single-cell suspensions were incubated with an Fc receptor blocker followed by staining with different fluorochrome-conjugated antibodies as described in Supplementary Table-1 [5]. After staining, cells were analyzed by FACS Fortessa using CellQuest software (BD Biosciences). t-distributed stochastic neighbor embedding (t-SNE) plot analysis was performed using FlowJo v10 software as described previously [30].

### Data mining and computational analysis

The Caldas (2007) [31] and Chin (2006) [32] datasets were obtained via the Xena Browser [33] and imported into the R version 3.5.3. Heat maps of gene expression compared to staging, size, and grade were developed using heatmap R package version 1.0.12 with expression values converted to ranks for improved visualization. The effect of S100A7 and cPLA2 mRNA expressions level on the OS probability in IM subtype of breast cancer patients was analyzed and the Kaplan-Meier plots were generated by the Kaplan-Meier Plotter [34]. The differential expression and correlation of S100A7 and cPLA2 were also analyzed by using different databases that include gene expression database of normal and tumor tissues version 2 (GENT2) and Search-Based Exploration of Expression Compendium (SEEK) databases. GENT2 provided a user-friendly gene expression search platform for analyzing the differential gene expression patterns across different normal and tumor tissues assembled from public gene expression data sets [35], while SEEK is a computational gene co-expression search engine.

### Immunoblotting and small interfering RNAs

Cell lysates were analyzed by immunoblotting as described earlier [36–38]. cPLA2 specific siRNA was purchased from Dharmacon and knockdown was achieved as described earlier [39].

### Immunohistochemistry (IHC), immunofluorescence (IF), and ELISA

IHC and IF on formalin-fixed sections were performed as described earlier [40]. Antibodies used are mentioned in Supplementary Table-1. The staining of TMAs was graded as previously described [38]. Human S100A7 and PGE2 ELISA kits were purchased from the MyBioSource and Novus Biologicals respectively and the assay was performed as per the manufacturer’s instructions. Plasma samples were stored at − 80 °C until analysis and processed for downstream study as described earlier [41].

### Cancer patient data analysis

Tissue microarrays (TMAs) for invasive breast cancer (BR1002b) were obtained commercially from US Biomax, Inc. (Rockville, MD). The clinicopathological detail of this TMA was provided in Supplementary Table-2. Normal and breast cancer patients’ frozen blood plasma samples were procured from TCC-OSUCCC after getting Institutional Review Board (IRB) approval (2019C0021). IHC profiler software was used to analyze the expression of different proteins. The infiltration of CD163^+^ M2-TAMs was analyzed by using ImageScope software. Non-parametric tests were performed to calculate the *p* values. Spearman rho’s correlation was also performed to calculate the correlation between different protein expressions and infiltration of CD163^+^ M2-TAMs.

### Statistical analysis

For continuous variables, two-sample *t*-tests were used if two groups were compared, and One-way ANOVA was used when more than two groups were compared. Non-parametric tests were also used to calculate the *p* values for comparing the clinical data of more than two groups. Spearman’s rho correlation analysis was used to calculate the correlation coefficient and p values. For the TCGA dataset using the Oncomine database, ****P < 0.0001 cut-off value was used for calculating statistical significance as described earlier [42, 43]. * indicates *P* < 0.05; ** indicates *P* < 0.01; *** indicates *P* < 0.001; **** indicates P < 0.0001; ns is non-significant.

## Results

### High co-expression of S100A7 and cPLA2 correlates with poor prognosis

**Fig. 1:**
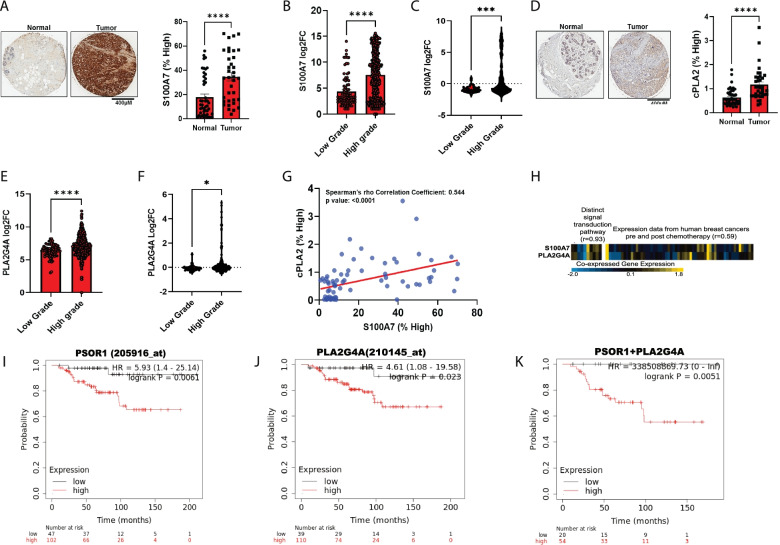
High co-expression of S100A7 and cPLA2 (PLA2G4A) is associated with poor clinical prognosis in breast cancer. Representative immunohistochemistry (IHC) images of (**A**). S100A7 and (**D**). cPLA2 proteins in malignant breast tumor samples (*n* = 36) and normal tissues (*n* = 46) [source: US Biomax]. Percent high-positive cells were quantified (right). Non-parametric test (Mann-Whitney U test) was applied. Relative mRNA expression (log2 fold change) of S100A7 and cPLA2 (PLA2G4A) were analyzed in low (*n* = 82) and high grades (*n* = 450) of breast tumor tissues using (**B** & **E**). GENT2 database (http://gent2.appex.kr/gent2/) and (**C** & **F**). also in low (*n* = 39) and high (*n* = 183) grades of breast cancer samples using Caldas & Chin datasets (*n* = 222). t test was used to calculate the *p* values (**G**). Correlation analysis between S100A7 and cPLA2 protein levels (% high positive) in a tumor tissue microarray (TMAs) of malignant breast cancer patients (*n* = 74) (**H**). Heatmap showing the correlation of S100A7 and cPLA2 in distinct signal transduction pathways (correlation coefficient = 0.93; NCBI GEO dataset: GSE39965) and expression in breast tumor tissues of pre-and post-chemotherapy (correlation coefficient = 0.59; NCBI GEO dataset: GSE18728). The pre and post chemotherapy analysis of baseline gene expression comprised baseline samples from 28 breast cancer patients which include17 non-responders and 11 responders. Analyses of gene expression changes involved paired baseline and post-cycle one specimens from 14 patients; of these 8 patients were non-responders and 6 patients were responders. (source: SEEK database). KM-plotter survival analysis (gene chip) of (**I**). S100A7 (PSOR1) alone, (**J**). cPLA2 (PLA2G4A) alone and (**K**). Combined S100A7 and cPLA2 (analysis include only patients with high median expression of genes) mRNAs expression with overall survival (OS) probability of immunomodulatory (IM) subtype of breast cancer patients. ns: non-significant, *P < 0.05, ** *P* < 0.01, *** *P* < 0.001, **** *P* < 0.0001. scale bar: 300 μm

Firstly, we analyzed the clinical significance of S100A7 and cPLA2 (PLA2G4A) in invasive breast cancer using publically available breast cancer datasets and human breast cancer tissue microarray (TMAs) by immunohistochemistry analysis. We discovered a significantly higher level of S100A7 and cPLA2 proteins in tumor tissues compared to normal breast tissues (Fig. 1A & D). We also observed significantly higher expression of both S100A7 and cPLA2 proteins in high-grade malignant breast tumors compared to the low-grade and normal tissues (Supplementary Fig. 1A-C). Next, we evaluated the relative mRNA expression of S100A7 and cPLA2 in low and high-grade breast tumor tissues using different publically available clinical datasets. Using the GENT2 database, we observed that higher-grade invasive breast tumors show increased expression of S100A7 and PLA2G4A compared to low-grade tumors (Fig. 1B & E). Further, we evaluated these results in independent cohorts of breast cancer patients. For this, we analyzed the differential expression of S100A7 and cPLA2 genes across different stages, grades, and tumor sizes of breast cancer patients using publically available Caldas and Chin datasets (Supplementary Fig. 1D). In agreement with our previous results, we observed that S100A7 and PLA2G4A revealed significantly higher expression in high-grade tumors compared to low-grade breast tumor tissues (Fig. 1C & F). Next, we evaluated the correlation between S100A7 and cPLA2 at the protein level using the same breast cancer patient samples. Interestingly, we found a significant positive correlation (Spearman’s rho correlation coefficient = 0.544) between S100A7 and cPLA2 (Fig. 1G). We further tested the correlation between S100A7 and PLA2G4A gene expression in breast cancer patients using the SEEK database (Search-Based Exploration of Expression Compendium). We observed a significant positive correlation between S100A7 and PLA2G4A in distinct signal transduction pathways (r = 0.93) and among different breast tumor samples from pre-and post-chemotherapy (r = 0.59) (Fig. 1H). Furthermore, we analyzed the correlation of S100A7 and PLA2G4A using the cBioPortal for cancer genomics database, which contains a large number of breast cancer samples of different subtypes. We discovered that S100A7 showed a significant positive correlation with PLA2G4A in breast cancer patients, mainly in breast invasive carcinoma (Supplementary Fig. 1E). We also evaluated the prognostic significance of S100A7 and PLA2G4A alone or in combination and overall survival (OS) probability of immunomodulatory (IM) subtype of breast cancer patients using the Kaplan Meier (KM)-plotter [Breast cancer] tool (gene chip). The KM plotter analysis revealed that alone or combined higher expression of both these genes had high hazard ratios and poor OS probability among the IM subtype of breast cancer subjects (Fig. 1I-K). Interestingly, we sought to analyze the clinical significance of S100A7 and cPLA2 in relapse-free survival at 5 years in breast cancer patients treated with any chemotherapy using the ROC (receiver operating characteristics) plotter database. ROC plotter database is capable to link gene expression and response to therapy using transcriptome-level data [44]. We discovered that S100A7 and cPLA2 showed significantly decreased expression in responder cohorts as compared to non-responder breast cancer patients with the area under curve (AUC) values of 0.571 (205916_at; Mann-Whitney test *p*-value:0.0076) and 0.555 (210145_at; Mann-Whitney test p-value:0.037) respectively (Supplementary Fig. 2A & B). Finally, we also evaluated the differential expression of these two genes in normal and different hormonal subtypes of breast cancer patients using TISIDB and Oncomine databases. We observed that only the basal subtype of breast cancer showed significantly high levels of S100A7 (p-value = 1.81e-22) and PLA2G4A (p-value = 1.45e-54) as compared to normal subjects (Supplementary Fig. 2C & D). Interestingly, the expression of both these genes was significantly high specifically in the TNBC subtype compared to another molecular status (Supplementary Fig. 2E & F). TNBC is frequently used as a surrogate for classifying the invasive basal breast cancer subtype and basal-like TNBC has been reported to be associated with poor clinical outcomes. Taken together, these findings suggest that S100A7 and cPLA2 are highly expressed and positively correlated with high-grade breast tumors. In addition, the coexpression of S100A7 and cPLA2 with poor overall survival for breast cancer patients, especially in aggressive IM and basal-like TNBC.

### cPLA2 inhibition attenuates PGE2 production in breast cancer cells

**Fig. 2:**
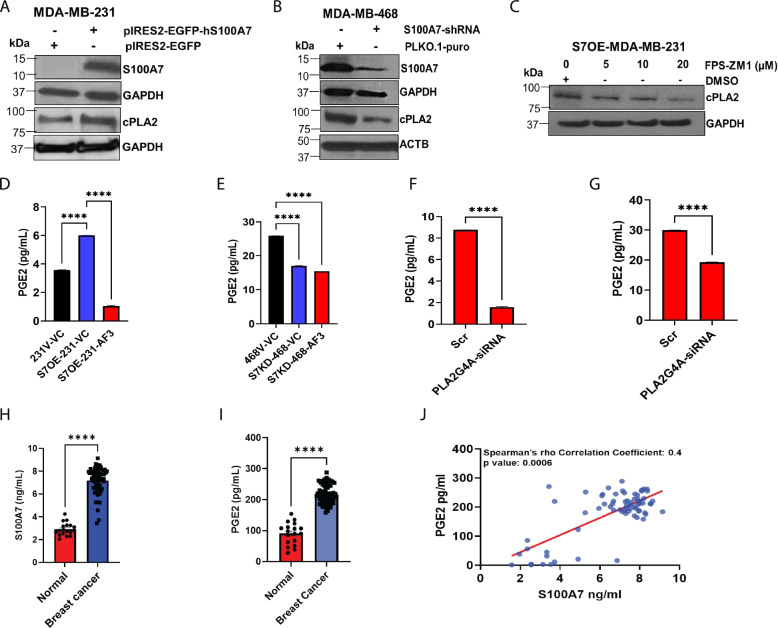
S100A7 regulates cPLA2-mediated PGE2 generation in breast cancer. Immunoblot analysis of S100A7 and cPLA2 proteins in (**A**). MDA-MB-231 vector (231 V) and S100A7 overexpression (S7OE-231) cells (**B**). MDA-MB-468 vector (468 V) and S100A7 knockdown (S7KD-468) cells (**C**). Immunoblot analysis of cPLA2 in S7OE-231 cells treated with vehicle control (DMSO) or RAGE inhibitor (FPS-ZM1) for 24 h. The bar diagram showing the levels of PGE2 (pg/mL) in conditioned medium (CM) harvested from (**D**). 231 V and S7OE-231 cells, and (**E**). 468 V and S7KD-468 either treated with vehicle control (VC) or AACOCF3 (AF3). Analysis of PGE2 (pg/mL) in CM of (**F**) S100A7-overexpressing and (**G**). MDA-MB-468 cells transiently transfected with scramble control (Scr) or PLA2G4A-siRNA. Estimation of (**H**). S100A7 (ng/mL) and (**I**). PGE2 (pg/mL) in blood plasma samples of normal subjects (n = 18) and breast cancer patients (*n* = 62) (**J**). Correlation between blood plasma level of S100A7 (ng/ml) and PGE2 (pg/ml) in breast cancer patients (*n* = 70). ns: non-significant, *P < 0.05, ** P < 0.01, *** P < 0.001, **** P < 0.0001. t test and one way ANOVA was used for calculating statistical significance

To further interrogate the correlation and determine the role of S100A7 in regulating cPLA2 expression and its downstream signaling, S100A7 overexpressing/downregulated breast cancer cells were analyzed. For this study, we first analyzed the expression of S100A7 in MDA-MB-231 and MDA-MB-468 cell lines and we observed that S100A7 is only expressed in MDA-MB-468 cells at basal condition (Supplementary Fig. 2G.). Therefore, we downregulated S100A7 in an MDA-MB-468 cell line, while we overexpressed S100A7 in MDA-MB-231 cells. We observed that S100A7 overexpression enhances cPLA2 expression in MDA-MB-231 cells (Fig. 2A), whereas S100A7 downregulation reduced the cPLA2 expression in MDA-MB-468 cells (Fig. 2B). In our previous study, we have shown that S100A7 mediates its effect by directly binding to the RAGE receptor in breast cancer cells [45]. In this study, we observed that exogenous supplementation of hS100A7 recombinant protein in S100A7 deficient MDA-MB-231 cells that express RAGE caused increased cPLA2 expression (Supplementary Fig. 2H). Therefore, we next tested the effect of RAGE inhibition on cPLA2 levels in S100A7 overexpressing MDA-MB-231 cells. The pharmacological inhibition of RAGE, using the FPS-ZM1 inhibitor, showed a dose-dependent reduction of cPLA2 expression in S100A7 overexpressing MDA-MB-231 cells (Fig. 2C).

Next, we explored the downstream mechanistic pathway regulated by the S100A7/cPLA2 axis in metastatic breast cancer cells. We observed that S100A7 overexpression significantly increases PGE2 levels, while the treatment of cPLA2 inhibitor (AACOCF3) led to reduced PGE2 secretion in MDA-MB-231 cells (Fig. 2D). In addition, the S100A7 knockdown reduced PGE2 generation that was not further significantly reduced by AACOCF3 in MDA-MB-468 cells (Fig. 2E). We also discovered that cPLA2 knockdown in S100A7 expressing breast cancer cells significantly reduced the PGE2 level (Fig. 2F & G) (Supplementary Fig. 2I & J). To further determine the clinical significance of S100A7 in regulating the PGE2 titer in breast cancer patients, we analyzed the titer of S100A7 and PGE2 and their correlation in breast cancer plasma samples. We discovered that the level of both S100A7/PGE2 was significantly higher in breast cancer patients as compared to normal subjects (Fig. 2H & I). We also observed a significant positive correlation (Spearman’s rho correlation coefficient = 0.4) between circulating S100A7 and blood PGE2 in breast cancer patients (Fig. 2J). Overall, our data suggest that cPLA2 inhibition suppressed S100A7-induced PGE2 generation by breast cancer cells.

### cPLA2 inhibition decreases S100A7-induced tumor burden in orthotopic and spontaneous breast cancer mouse models

**Fig. 3:**
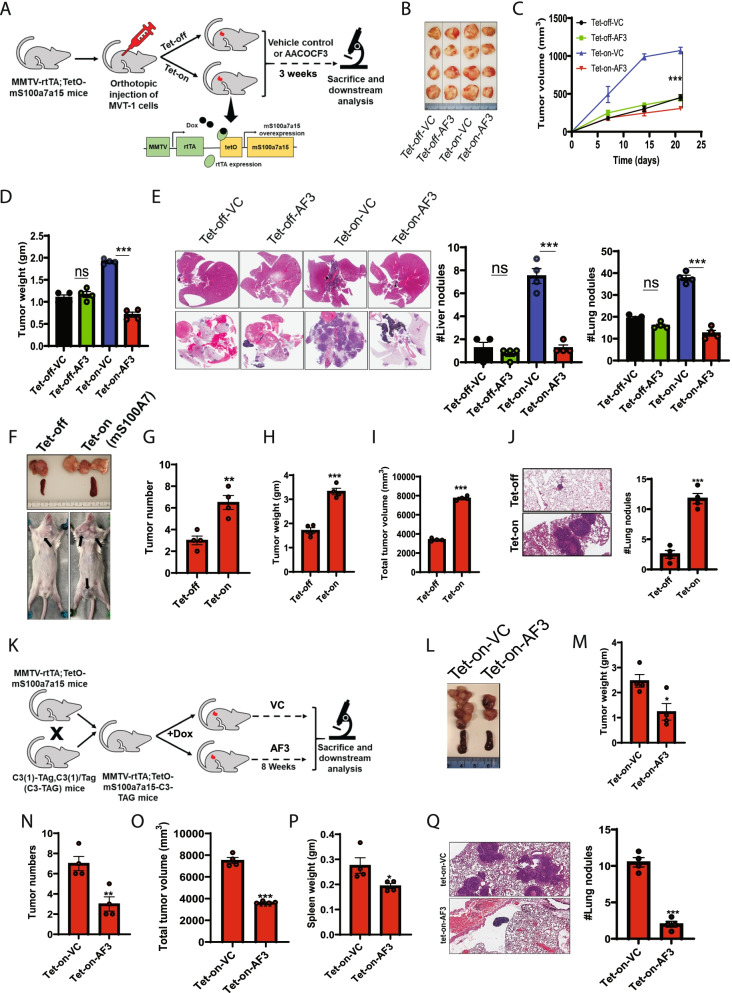
Inhibition of cPLA2 reduces S100A7-enhanced breast tumor growth and metastasis in MMTV-rtTA;TetO-mS100a7a15 bi-transgenic mice model (**A**). Schematic representation showing the generation and treatment of the doxycycline (DOX) inducible mS100a7a15 bi-transgenic (Tet-O, tet operator) MVT1 tumor-bearing mouse model. In brief, 1X10^5^ MVT1 cells into the 4th mammary gland (MG) of the bi-transgenic mice. Mice were fed with either 1 g/kg.bt. doxycycline diet (Tet-on; n = 8) or normal diet (Tet-off; n = 8). After the onset of palpable tumors, each group was divided into 2 subgroups (n = 4 each) and were treated with VC or AF3 (5 mg/kg.bt) intraperitoneal (i.p.) twice a week for 3 weeks. Tumor volume was measured once a week in these mice. After 21 days, the tumors were harvested from these mice and weighed (**B**). Representative photographs of tumors dissected from different experimental groups (**C**). Graph showing the tumor volume (mm^3^) and (**D**). tumor weight (gm) of each experimental group treated with VC and AF3 (**E**). Representative image of H&E staining of metastatic nodules in the liver (top) and lung (bottom) of VC and AF3 treated mice. Bar diagram represents the means ±SEMs of four replicates (**F**). Representative image showing the tumors harvested from Tet-off (normal diet) and Tet-on (DOX diet) groups with MMTV-rtTA;TetO-mS100a7a15-C3-TAG genotype. Graph showing the (**G**). the total number of tumors, (**H**). tumor weight (gm) (**I**). total tumor volume (mm^3^) and (**J**). the number of lung nodules in Tet-off and Tet-on groups (**K**). Schematic approach showing the treatment of spontaneous breast cancer model of mS100a7a15 overexpressing (MMTV-rtTA;TetO-mS100a7a15-C3-TAG) mice. MMTV-rtTA;TetO-mS100a7a15-C3-TAG female mice (6 weeks old) were fed with DOX diet (Tet-on) and after the onset of palpable tumors, mice were either treated with VC or AF3 (5 mg/kg.bt) intraperitoneal (i.p.) twice in a week for 8 weeks. Tumor volumes were measured externally by using Vernier caliper. At the endpoint, mice were sacrificed and tumors and other organs were harvested for downstream analysis (**L**). Photomicrographs of tumors harvested from Tet-on mice treated with VC or AF3. Graph showing the (**M**). tumor weight (gm), (**N**). the total number of tumors, (**O**). total tumor volume (mm^3^) and (**P**). Spleen weight (gm) and (**Q**). the number of lung nodules in Tet-off and Tet-on groups. The graphs indicate the means ±SEMs of four replicates. ns: non-significant, *P < 0.05, ** *P* < 0.01, *** *P* < 0.001. t test and one way ANOVA was used for calculating statistical significance

To evaluate the preclinical significance of cPLA2 inhibition in S100A7-overexpressing mammary tumors, we assayed the inhibitory effect of AACOCF3 on orthotopic and spontaneous breast cancer mouse models. mS100a7a15 is a paralog of human S100A7, and we have generated a doxycycline (DOX)-inducible bi-transgenic MMTV-mS100a7a15 mouse model [5]. These mice overexpress mS100a7a15 in the mammary glands in presence of DOX. For this study, we injected MVT-1 cells in the mammary fat pad of these mice and after the formation of palpable tumors, we divided these mice into 4 different experimental groups: (a) without DOX (normal diet) with vehicle control (Tet-off-VC), (b) without DOX (normal diet) with AACOCF3 (Tet-off-AF3), (c) DOX diet with vehicle control (Tet-on-VC), and (d) DOX diet with AACOCF3 (Tet-on-AF3) (Fig. 3A). Surprisingly, only mice overexpressing mS100a7a15 respond to cPLA2 inhibitor and showed significantly decreased tumor burden after the treatment of AACOCF3 compared to control animals (Fig. 3B-D). Increased expression of mS100a7a15 was confirmed by immunofluorescence assay in MVT-1 tumors under DOX diet (Tet-on) than mice on a normal diet (Tet-off) (Supplementary Fig. 3A). Visceral organ analysis revealed that cPLA2 inhibition significantly reduced lung and liver metastases, preferentially in Tet-on-AF3 group (Fig. 3E) (Supplementary Fig. 3B). Importantly, we also observed that cPLA2 inhibition more drastically reduced the size and weight of the spleen from Tet-on (mS100a7a15-high) group compared to the Tet-off group (Supplementary Fig. 3C). The spleen plays an essential role in neoplastic growth by serving as a reservoir of many biological factors during the different stages of tumor growth [46] and has been reported as an essential extramedullary site that can unceasingly support tumor growth by increasing the infiltration of immunosuppressive myeloid cells into the tumor [47].

We also investigated the inhibitory potential of AACOCF3 on a spontaneous C3(1)/SV40 T/t-antigen transgenic mouse model of human triple-negative breast cancer [48]. We cross-bred MMTV-rtTA;TetO-mS100a7a15 mouse with C3(1)-TAg,C3(1)/Tag (C3-Tag) mouse and animals with C3-Tag;MMTV-rtTA;TetO-mS100a7a15 genotypes were maintained in presence or absence of DOX diet for eight weeks. Mice fed with the DOX diet showed significantly higher tumor burden and pulmonary metastasis compared to mice on a normal diet (Fig. 3F-J). Next, we evaluated the anti-tumor effect of AACOCF3 treatment on C3-Tag;MMTV-rtTA;TetO-mS100a7a15 mice fed with DOX diet. We found that AACOCF3 treatment significantly reduced S100A7 enhanced tumor burden and lung metastasis (Fig. 3K-Q). Altogether, our in-vivo studies suggest that cPLA2 inhibition tends to be a promising strategy against S100A7 overexpressing metastatic mammary tumors.

### S100A7/cPLA2/PGE2 signaling enhances immunosuppressive tumor microenvironment

Breast cancer iTME constituents possess the ability to reprogram tumor growth and distant metastasis; hence, a better understanding of the iTME would help in designing effective approaches for efficient targeting of S100A7-overexpressing metastatic breast cancers. Hence, we evaluated the functional significance of cPLA2 inhibition in regulating the recruitment of different myeloid cells for generating an iTME. We demonstrated that high expression of the cPLA2 gene positively correlated with the increased abundance of tumor-associated macrophages (TAMs) and myeloid-derived suppressor cells (MDSCs) in invasive breast tumor tissues (Supplementary Fig. 4A-B).

**Fig. 4:**
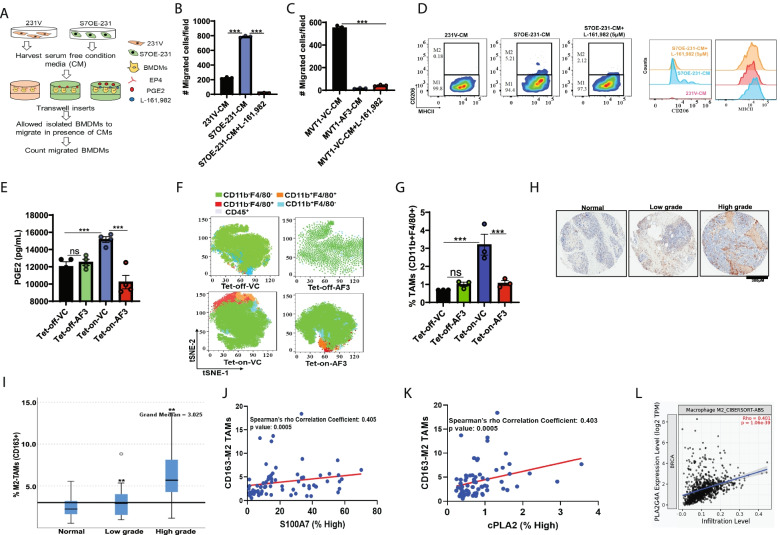
S100A7/cPLA2 signaling enhances the infiltration of tumor-associated macrophages (TAMs) through increased secretion of PGE2 (**A**). Schematic representation showing the treatment of conditioned media (CM) derived from MDA-MB-231 vector (231 V) and S100A7 overexpressing cells (S7OE-231) in presence or absence of PGE2 receptor, EP4 inhibitor on migration of bone marrow derived macrophages (BMDMs). Bar diagram shows the number of migrated BMDMs per field stimulated with CM derived from (**B**). 231 V and S7OE-231, (**C**). MVT1 cells treated with VC or AF3 or BMDMs preincubated with EP4 receptor antagonist (5 μM L161,982) for 2 h before stimulation with CM of MVT1 cells. Overnight migration using transwell inserts was performed. The bars indicate the means ± SEMs of 3 replicates (**D**). Flow cytometric analysis of MHC-II and CD206 in BMDMs cultured in CM of 231 V and S7OE-231 cells or BMDM preincubated with EP4 receptor antagonist (5 μM L161,982) for 2 h before stimulation with CM of S7OE-231 cells (**E**). Quantitative analysis of blood PGE2 (pg/mL) in MVT1 tumor-bearing Tet-off (normal diet) and Tet-on (DOX diet) mice treated with VC or AACOCF3 (AF3) (**F**). CD11b^+^F4/80^+^ TAMs (out of CD45^+^ cells) in tumors harvested from MVT1 tumor-bearing Tet-off (normal diet) and Tet-on (DOX diet) mice treated with VC or AF3 (**G**). The bar diagram indicates the means ±SEMs of three replicates (**H**). Representative IHC images of CD163^+^ M2-TAMs in adjacent normal, low-grade, and high-grade invasive breast cancer specimens (source: Biomax) (**I**). Box plot showing the percentage (%) of CD163^+^ M2-TAMs in normal, low-grade, and high-grade invasive breast cancer specimens. Non-parametric test (Independent-Samples Median Test) for comparing high grade to low grade and adjacent normal specimens of breast cancer patients. Bonferroni correction was applied for multiple tests. Spearman rho’s correlation coefficient analysis between abundance of CD163^+^ M2-TAMs with protein levels (% high positive) of (**J**). S100A7 and (**K**). cPLA2 in TMAs of breast cancer patients (*n* = 70) (source: Biomax) (**L**). Spearman rho’s correlation coefficient analysis of cPLA2 mRNA (PLA2G4A) expression with infiltration of M2-TAMs in breast cancer patients (source: TIMER2.0; http://timer.cistrome.org/). ns: non-significant, *P < 0.05, ** *P* < 0.01, *** *P* < 0.001. One way ANOVA was used for multiple group comparisons

PGE2 has been reported to play an important role in the generation of an iTME by modulating immunosuppressive myeloid cells (TAMs and MDSCs) in different human malignancies [16]. Here, we investigated the role of the S100A7/cPLA2/PGE2 axis in affecting bone marrow-derived macrophages (BMDMs) migration and plasticity by using cPLA2 and EP4 (PGE2 receptor) inhibitors. We observed that S100A7 overexpression significantly increased the migratory potential of BMDMs and treatment of cPLA2 inhibitor (AACOCF3) or EP4 inhibitor (L-161,982) drastically reduced the S100A7/PGE2-induced migration of BMDMs (Fig. 4 AC) (Supplementary Fig. 4C & D). Furthermore, BMDMs were treated with EP4 inhibitor or incubated with conditioned media of AACOCF3 treated S100A7 expressing breast cancer cells. We observed a decreased differentiation of BMDMs into CD206^+^MHC-II^low^ M2-like macrophages in presence of cPLA2 or EP4 inhibition (Fig. 4D) (Supplementary Fig. 4E).

Consistent with our in-vitro observations, we found that AACOCF3 significantly reduced blood PGE2 levels as well as the abundance of TAMs in the tumor and spleen of tumor-bearing S100A7-overexpressing mice (Fig. 4E-G) (Supplementary Fig. 5A-C). To further analyze the clinical impact of S100A7/cPLA2 signaling in modulating the host immunosuppressive response, we investigated the correlation of these two proteins with the abundance of tumor-promoting CD163^+^ M2-TAMs using TMAs of invasive breast cancer patients. Similar to our previous results, we discovered that different grades of malignant breast tumors revealed a significantly increased abundance of CD163^+^ M2-TAMs compared to normal breast tissues (Fig. 4H & I) and also revealed a significant positive correlation with increased expression of S100A7 and cPLA2 proteins (Fig. 4J & K). Moreover, cPLA2 gene expression is also significantly positively correlated with CD163^+^ M2-TAMs in breast cancer patients (Fig. 4L). Additionally, KM plotter analysis also indicates that high expression of S100A7 with enriched macrophage population significantly associated with poor RFS probability for breast cancer patients, while high S100A7 expression with decreased macrophage showed non-significant clinical outcome (Supplementary Fig. 5D).

**Fig. 5:**
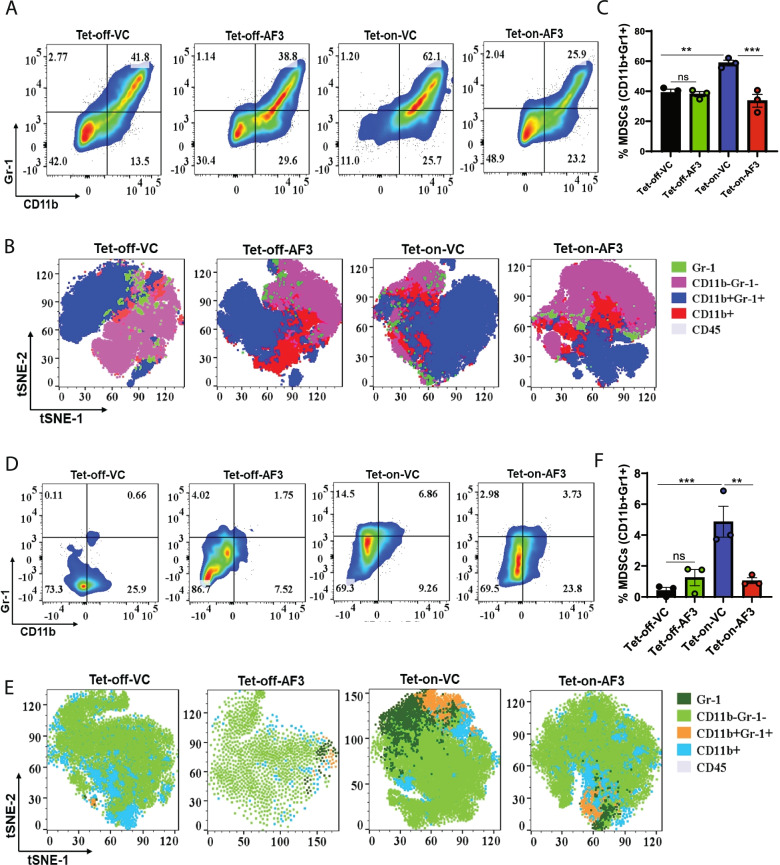
cPLA2 inhibitor decreases S100A7-mediated recruitment of myeloid-derived suppressor cells (MDSCs) in MMTV-rtTA;TetO-mS100a7a15 bi-transgenic mice model (**A**). Flow cytometric and (**B**). t-SNE plot analysis of CD11b^+^Gr-1^+^ MDSCs (out of CD45^+^) in spleens harvested from Tet-off and Tet-on mice treated with VC or AF3 (**C**). Bar diagram represents the means ±SEMs of three replicates (**D**). Flow cytometric and (**E**). t-SNE plot analysis of MDSCs in tumors harvested from Tet-off and Tet-on mice treated with VC or AF3 (**F**). Bar diagram represents the means ±SEMs of three replicates. ns: non-significant, *P < 0.05, ** P < 0.01, *** P < 0.001. One way ANOVA was used for multiple group comparisons

We also investigated the effect of cPLA2 inhibition on S100A7-mediated recruitment of MDSCs using our mS100a7a15 overexpressing mice. Interestingly, only tumor-bearing mS100a7a15-overexpressing bi-transgenic mice responded to cPLA2 inhibitor and showed reduced recruitment of MDSCs both in spleen and tumors (Fig. 5A-F). Different subsets of MDSCs promote the development of tumors and metastasis [49]. We observed that the cPLA2 inhibitor selectively reduced the abundance of granulocytic Ly6G^+^ MDSCs in the spleen of mS100a7a15-overexpressing mice (Supplementary Fig. 6A-D). In brief, these results suggest that the S100A7/cPLA2/PGE2 signaling cascade generates an iTME through modulating the recruitment and plasticity of different immunosuppressive myeloid cells, especially TAMs in metastatic breast cancer, and therefore, disrupting this signaling mechanism may heighten the anti-tumor immune response.

### cPLA2 inhibition increases the infiltration of T lymphocytes in tumor

The anti-tumor effect of any chemotherapeutic drugs depends on two facets of cancer biology, first, the direct killing of tumor cells, and secondly is their ability to enhance the CD4^+^ and CD8^+^ tumor-infiltrating lymphocytes (TILs)-mediated immune response [50, 51]. Therefore, in this study, we utilized CODEX multiplexed imaging technique, which helps us to explore the functional significance of targeting the S100A7/cPLA2 signaling axis on abundance, cell surface expression, and interactions of different subsets of CD4^+^ and CD8^+^ TILs by using our tumor-bearing MMTV-mS100a7a15, a bi-transgenic mouse model. CODEX is a recently developed highly multiplexed imaging technique that enables the analysis of more than 56 proteins in a single section of tissue through an iterative imaging process [52, 53]. Spatial interaction of host-tumor cells with immune cells and expression levels of different markers can be easily quantified using this multiplexed technique. Using known cell surface markers, this technique has proved to be particularly useful in profiling the precise immune cell populations in healthy and diseased tissues [52, 53]. As we discovered that cPLA2 inhibitor treatment was significantly effective only in the DOX-inducible mouse model of mS100a7a15, therefore in this study, we investigated the anti-tumor effect of AACOCF3 on immune response mediated through CD4^+^ and CD8^+^ TILs in mS100a7a15 overexpression group using CODEX.

**Fig. 6:**
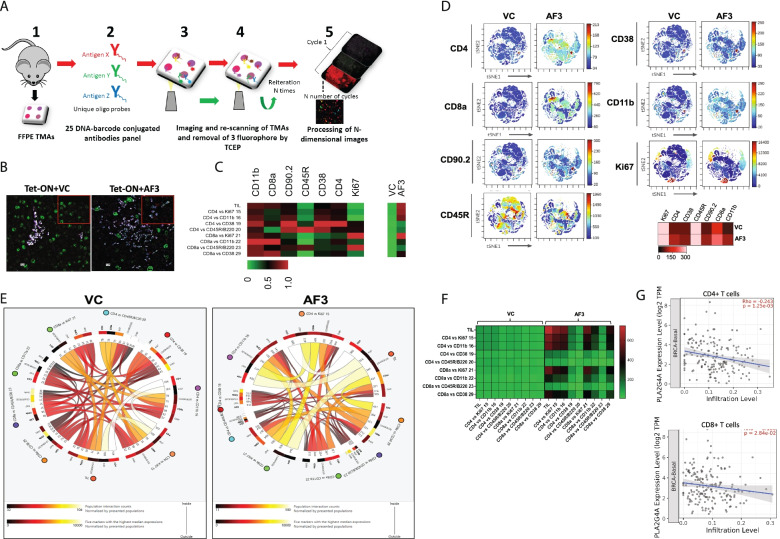
cPLA2 inhibitor increases the infiltration of proliferative activated CD4/CD8 tumor-infiltrating T lymphocytes (TILs) in TME of tumor bearing S100A7 overexpressing mice (**A**). Schematic diagram of CODEX workflow showing the different steps of FFPE TMAs staining (**B**). Seven-color image of FFPE TMA prepared from of orthotopic syngeneic breast cancer model of mS100a7a15 bi-transgenic mouse model and imaged using a 25 DNA-barcode conjugated antibodies panel. 7 colors show the following markers: red- CD8a, yellow- CD45R, cyan- CD11b, White- CD90.2, blue- CD4, magenta- CD38, and green- Ki67 (**C**). Heatmap showing the average strength of cell-type to cell-type interaction for different clusters of vehicle control (VC) and cPLA2 inhibitor (AF3) treated mS100a7a15 overexpression mouse model (**D**). t-SNE and heatmap showing the expression of different markers associated with proliferating and activated CD4+ and CD8+ T cells (**E**). Circos plot and, (**F**). heat map showing the cell interaction networks of different subsets of proliferating and activated CD4+ and CD8+ T cells in VC and AF3 treated groups. The thickness of interacting connection associates with the number of contacts, size of the node represents the number of cells per group. Scale bar in heat map showing the interaction strength of different connecting nodes (**G**). Spearman rho’s correlation coefficient analysis of cPLA2 mRNA (PLA2G4A) expression with infiltration level of CD4 and CD8 T cells in invasive basal-like breast cancer patients (source: TIMER2.0; http://timer.cistrome.org/)

CODEX was performed using validated antibodies for mouse tumor tissue samples (Fig. 6A) [53]. Since, in our study, CODEX was performed on FFPE breast tumor tissues, we focused our analysis and interpretations on those T cells markers, which showed positive signals in our samples. The in-depth quantitative and cluster analysis revealed that the AACOCF3 treatment increased the infiltration of proliferating (Ki-67^+^) and activated (CD11b/CD45R/CD38/CD90.2) CD4^+^ and CD8^+^ TILs in breast TME (Fig. 6B & C; Supplementary Fig. 7). CD11b, CD38, CD45R, and CD90.2 are murine cell surface markers known to be associated with the activated status of T cells [54–59]. We also analyzed the cell populations expressing CD4, CD8a, CD90.2, CD45R, CD38, CD11b, and Ki-67 using t-SNE plot analysis and we found that AACOCF3 treatment increased the number of CD4, CD8a, CD90.2, CD38, and CD11b positive cells (Fig. 6D). The anti-tumor activity of TILs depends on mutual direct or indirect interaction of different subsets of T cells [60–62], therefore we investigated the effect of cPLA2 inhibition on the interaction of proliferating and activated TILs. We presented the cell-cell interaction map in circus and heatmap plots and we observed that AACOCF3 treatment revealed the highest degree of interaction among the different subsets of proliferating and activated CD4^+^ and CD8^+^ TILs (Fig. 6E & F). Finally, we explored the association of CD4^+^ and CD8^+^ T cells with cPLA2 (PLA2G4A) gene expression in invasive basal-like breast cancer patients using the TIMER database. Interestingly, we found that cPLA2 differential expression showed a significant negative correlation with CD4^+^ and CD8^+^ T cells infiltration (Fig. 6G). In brief, our results showed that the inhibition of S100A7/cPLA2 signaling could increase the infiltration of proliferating and activated CD4^+^ and CD8^+^ TILs in breast TME and may mount an enhanced anti-tumor immune response against aggressive metastatic breast cancer cells.

### cPLA2 inhibition attenuates tumor progression in humanized PDX mouse model by decreasing tumor-infiltrating immunosuppressive cells

**Fig. 7:**
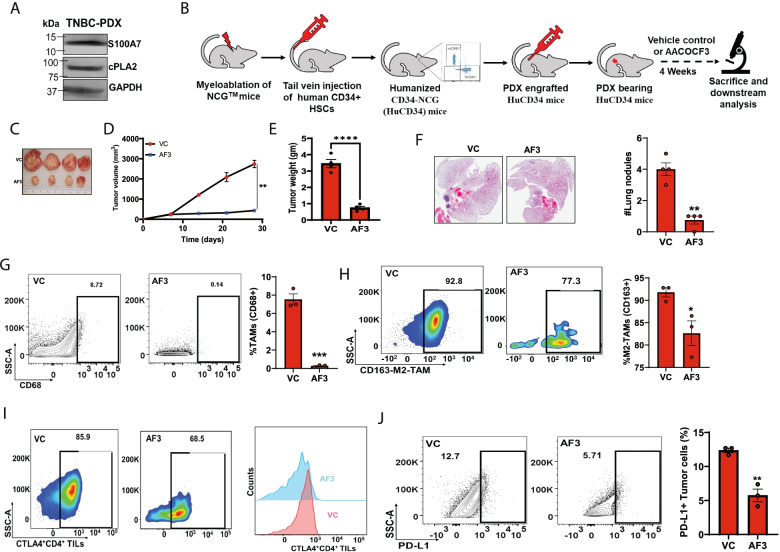
cPLA2 inhibition obstructs tumor growth and metastasis in Hu-PDX mice model by attenuating immunosuppressive cells (**A**). Immunoblot analysis of S100A7 and cPLA2 proteins in tumor lysate of breast cancer patient derived-xenograft (PDX) specimen. GAPDH was used as a loading control (**B**). Schematic diagram showing the methodology for generation and treatment strategy of humanized PDX (Hu-PDX) breast cancer model (**C**). Representative image of tumors harvested from VC or AF3 (5 mg/kg.bt) treated Hu-PDX mice model. Graph showing the (**D**). tumor volume (mm^3^) and (**E**). Tumor weight (gm) of VC or AF3 treated PDX mice model (**F**). H/E image of lung nodules in lungs harvested from VC or AF3 treated Hu-PDX groups. The graphs indicate the means ±SEMs of four replicates. Flow cytometric analysis of % (**G**). total CD68^+^ TAMs (out of EpCAM^−^), (**H**). CD163^+^M2-TAMs (out of EpCAM^−^CD68^+^ macrophages) and (**I**). Exhausted CTLA^+^ CD4 T (out of EpCAM^−^CD14^−^CD3^+^CD4^+^) cells (**J**). PD-L1^+^ EpCAM^+^ tumor cells harvested from VC or AF3 treated Hu-PDX groups. The graphs indicate the means ±SEMs of three replicates. ns: non-significant, *P < 0.05, ** P < 0.01, *** P < 0.001. t test was used for statistical significance

To determine if the S100A7/cPLA2 signaling axis could be exploited as a potential therapeutic target against metastatic breast cancer, we analyzed the clinical utility of cPLA2 inhibitor against breast tumor growth and metastasis using the Hu-PDX mouse model. Hu-PDX breast cancer mouse models are emerging tools for evaluating the efficacy of novel drugs as well as immunotherapies on tumor growth, immune response, metastasis as they recapitulate the histological characteristics of the original tumors. First, we confirmed the expression of both S100A7 and cPLA2 in tumor lysate from the PDX specimen (Fig. 7A). We assessed the anti-tumor activity of AACOCF3 by using the Hu-PDX model (Fig. 7B) and found that AACOCF3 treatment significantly reduced tumor growth and pulmonary metastasis (Fig. 7C-F). We further explored the effect of cPLA2 inhibition on the immunosuppressive immune landscape of breast TME and observed that AACOCF3 treatment significantly reduced the recruitment of total TAMs and M2-TAMs (Fig. 7G & H). Interestingly, no significant change in the infiltration of CD8^+^ T cells while reduced infiltration of CD4^+^ T cells was detected (Supplementary Fig. 8A). Further, we analyzed the status of CD4^+^ T cells to predict the status of an adaptive anti-tumor immune response. We observed an increased CTLA-4 expression on CD4^+^ T cells in the control groups, which limits CD4^+^ T cell proliferation and its interaction with other immune cells. AACOCF3 treatment significantly reduced CTLA-4^+^ CD4^+^ T cells (Fig. 7I) while PD1^+^ CD4^+^ T cells were unaffected (Supplementary Fig. 8B). cPLA2 regulates the biosynthesis of PGE2 and PGE2 upregulates PD-L1 expression in different cell types [19, 63]. Therefore, we also analyzed the abundance of PD-L1^+^ tumor cells and found that AACOCF3 significantly decreased the expression of PD-L1 on tumor cells (Fig. 7J). Consequently, our preclinical data using Hu-PDX provide useful information for developing cPLA2 inhibitors against metastatic breast cancers.

## Discussion

Distant metastasis is one of the major clinical hurdles for the successful therapies of invasive breast cancer. Given the worst clinical prognosis and lack of established molecular targets in metastatic breast cancer patients, there is an utmost need for a greater understanding of the molecular mechanisms that enhance the metastatic potential of invasive breast cancer cells. This is important for developing better therapies for metastatic breast cancer patients, especially TNBC and basal like-breast cancer patients. S100A7-mediated signaling pathways promote inflammation, which contributes to aggressive breast tumor growth and metastasis [5, 45, 64–66]. Recent clinical data show that the copy number of the chromosomal region containing S100A genes, including S100A7, is amplified in cancer stem cells of TNBC and basal subtypes [67]. However, the S100A7-mediated downstream molecular mechanism which modulates iTME in metastatic breast cancer is not yet explored.

**Fig. 8:**
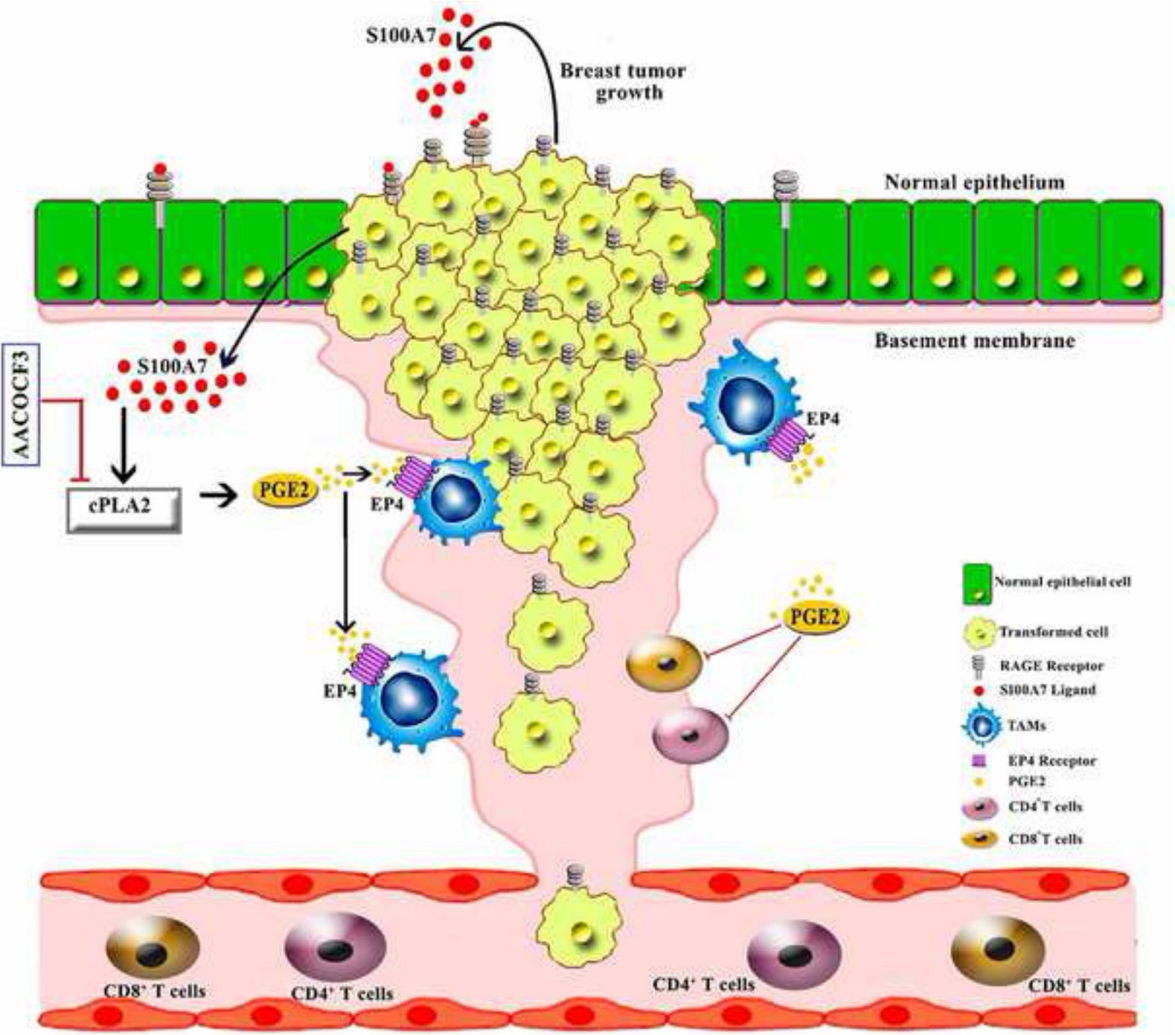
Schematic diagram highlighting S100A7/cPLA2 signaling cascade and its effect on tumor growth and metastasis of invasive breast cancer through modulating the tumor microenvironment (TME)

Here, we demonstrate that the S100A7/RAGE axis enhances cPLA2 expression in metastatic breast cancer cells (Fig. 8). Although S100A7 and cPLA2 individually are overexpressed in breast cancer [68, 69], no study has been performed to show their correlation in metastatic breast cancer. In our present study, Kaplan-Meier (KM) plotter analysis revealed that high co-expression of both S100A7 and cPLA2 correlates with decreased overall survival of breast cancer patients. In addition, we discovered that S100A7 expression strongly correlated with cPLA2 expression in metastatic breast cancer patients. We also observed that poorly differentiated high-grade breast tumors expressed a high level of both S100A7 and cPLA2 as compared to well-differentiated mammary tumors and their adjacent normal tissues. These studies indicate a positive correlation between S100A7 and cPLA2 in metastatic breast cancer patients and could be used as a potential prognostic marker for subsets of breast cancer patients.

We discovered that S100A7 expression regulates the production of PGE2 in breast cancer cells. We further demonstrated that cPLA2 inhibition caused reduced production of PGE2 only in S100A7 expressing breast cancer cells. Interestingly, a positive correlation between S100A7 and PGE2 levels was observed in breast cancer patient samples. Malignant breast tumors contain high levels of PGE2 [70]. This is the first line of evidence, which indicates that S100A7 positively correlated with PGE2 and that could be used as potential circulating biomarkers in metastatic breast cancer.

In various pre-clinical mouse models including the S100A7 overexpression bi-transgenic and Hu-PDX mouse models, we showed that inhibition of S100A7/cPLA2 signaling by using small molecule inhibitor against cPLA2 significantly reduced tumor burden and metastasis. cPLA2 inhibitors have shown improved efficacy in phase I and II clinical trials against psoriasis [22]. Psoriatic skin has been shown to express a higher level of Psoriasin (S100A7) and is characterized by dense infiltration of macrophages [71, 72]. Our studies also revealed that S100A7 and cPLA2 are positively correlated with the infiltration of CD163^+^ M2-TAMs in invasive breast tumor tissues. KM plotter analysis also revealed high expression of S100A7 with enriched macrophage populations significantly associated with the poor recurrence-free survival (RFS) of breast cancer patients. In contrast, high S100A7 expression with decreased macrophage density does not predict the patient outcome. Here, we demonstrate that cPLA2 inhibition drastically decreased the infiltration of TAMs in a syngeneic mS100a7a15 overexpressing breast cancer mouse model. In human breast tumors, infiltrating TAMs, which represent up to 50% of the tumor mass, correlate with poor prognostic features, higher tumor grade [73], and decreased disease-free survival [74, 75]. Higher TAM density is typically associated with a higher vascular density, suggesting an angiogenic role of TAMs in human tumors [76]. In this study, we further showed that S100A7/cPLA2 signaling elevated the PGE2 generation that increased migration and polarization of macrophages towards M2-type through the EP4 receptor of BMDM. Interestingly, we also found that inhibition of S100A7/cPLA2 signaling reduced PGE2 level in S100A7 overexpressing pre-clinical breast cancer mouse model. PGE2 has been shown to generate the iTME in different human malignancies by modulating the behavior of different immune cells [16]. Notably, PGE2/EP4 has also been reported to increase the expression of PD-L1 on MDSCs and TAMs [19].

TME is an essential part of the solid tumor mass and is considered a promising therapeutic target [77]. The presence of tumor-infiltrating lymphocytes (TILs) in the TME indicates mounting of an immune response against the tumor [78]. Recent studies have documented that CD4^+^ T cells are also effective at tumor rejection similar to CD8^+^ T cells [79, 80]. Therefore, we utilized a Hu-PDX mouse model to elucidate the effect of cPLA2 inhibition in modulating the tumor-infiltrating CD4^+^ and CD8^+^ lymphocytes. We found that pharmacological inhibition of cPLA2 reduced breast tumor growth and metastasis with a decreased abundance of only CTLA4^+^ CD4^+^ T cells and M2-TAMs. In addition, it also decreased the density of PD-L1^+^ cancer cells to evoke immune evasive mechanisms that prevent CD8^+^ T cell cytotoxicity [81]. Moreover, CODEX analysis of orthotopic mouse mammary tumors demonstrated that inhibiting S100A7/cPLA2 signaling increased the abundance of proliferating as well as activated CD4^+^ and CD8^+^ TILs. Taken together, our study also highlights the potential of cPLA2 inhibitors in increasing the sensitivity of breast cancer cells to existing immunotherapeutic agents.

## Conclusion

Our comprehensive studies using in-vitro assays, in-vivo mouse models, and patient samples showed that a novel cross-talk between S100A7 and cPLA2 enhances breast cancer growth and metastasis. We further showed that S100A7/cPLA2 signaling modulate TME by increasing the recruitment of immunosuppressive myeloid cells and reducing CD4^+^/CD8^+^ T cells. Our study provides usefulness in examining S100A7/cPLA2 expression as a potential prognostic marker for invasive and metastatic breast cancer patients. Furthermore, cPLA2 inhibitors could be used as a novel therapeutic drug for the treatment of metastatic breast cancer patients who harbor amplification or overexpression of the S100A7 gene. Overall, these studies have the potential to develop personalized therapy for metastatic breast cancer patients.

## Supplementary Information

**Additional file 1: Supplementary Table 1**: List of reagents and resources.

**Additional file 2: Supplementary Table 2**: Clinicopathological detail of Tissue microarray (BR1002b).

**Additional file 3: Figure S1**. Expression and correlation of S100A7 and cPLA2 in breast cancer types. (A). Representative S100A7 and cPLA2 immunohistochemistry (IHC) images of invasive breast cancer specimens. [source: US Biomax]. Box plot showing percent (%) high positive (B). S100A7 and (C). cPLA2 stained cells in normal (*n* = 46), low-grade (*n* = 15), and high-grade (*n* = 21) breast cancer specimens. Non-parametric test (Independent-Samples Median Test) was used to calculate *p* values. (D). Heat map analysis showing the differential expression of S100A7 and PLA2G4A across different grades, size, and stages of breast cancer patients using Caldas (*n* = 113) & Chin; (*n* = 109) datasets. (E). Correlation analysis of S100A7 and PLA2G4A was analyzed in different breast cancer types (*n* = 3380) using cBioPortal for Cancer Genomics. *P < 0.05*, *P < 0.01, ***P < 0.001.

**Additional file 4: Figure S2**. Effect of S100A7/cPLA2 gene expression on recurrence free survival of breast cancer patients. (A). ROC plotter analysis of S100A7 (205916_at; Mann-Whitney test *p*-value: 0.0076) and (B). cPLA2 (210145_at; Mann-Whitney test p-value: 0.037) for relapse free survival at 5 years after treatment with any chemotherapy in breast cancer patients. The graphs were plotted by using ROC plotter online database (http://www.rocplot.org/) with responder (*n* = 256) and non-responder (*n* = 220) breast cancer patients. Gene expression of (C). S100A7 and (D). PLA2G4A were analyzed in normal subjects (*n* = 137) and different breast cancer subtypes (Basal = 172; Her2 = 73, Luminal A = 508 and Luminal B = 191) using TISIDB database. (E). S100A7 (Reporter: A_23_P103310) and (F). PLA2G4A (Reporter: A_23_P11682) mRNA expressions were analyzed in triple negative (TNBC) and other biomarker status of invasive ductal breast carcinoma using TCGA breast cancer dataset of Oncomine database. [0 = no value or unidentified hormonal status (*n* = 297), 1 = HER2/ER/PR negative or TNBC (*n* = 46), 2 = other breast cancer biomarkers (*n* = 250)]. (G). Immunoblot analysis of S100A7 in MDA-MB-231 and MDA-MB-468 cells. (H). Effect of recombinant human S100A7 treatment (100 ng/ml for 24 h) on cPLA2 expression in MDA-MB-231 cells. Immunoblot analysis of cPLA2 in (I). S7OE-231 and (J). MDA-MB-468 cells transiently transfected with control or cPLA2-siRNA.

**Additional file 5: Figure S3**. Pharmacological inhibition of cPLA2 suppress the S100A7-mediated metastasis and gain in spleen weight in syngeneic orthotopic MMTV-rtTA;TetO-mS100a7a15 bi-transgenic mice model. (A). Immunofluorescence (IF) analysis of mS100a7a15 expression in tumor tissues harvested from MVT1 tumor-bearing mice fed with normal diet (Tet-off) or with doxycycline diet (Tet-on). (B). Representative images of livers (top) and lungs (bottom) harvested from Tet-off and Tet-on mice treated with VC or AF3. (C). Representative images of spleens harvested from Tet-off and Tet-on mice treated with vehicle control (VC) or 5 mg/kg.bt of AACOCF3 (AF3). Bar diagram represents the means of spleen weight (gm) ± SEMs of four replicates. * *P* < 0.05, *** *P* < 0.001. One way ANOVA was used for multiple group comparisons.

**Additional file 6: Figure S4**. S100A7/cPLA2 signaling regulates the recruitment and plasticity of myeloid cells in breast cancer. Correlation analysis of (A). Macrophage and (B). Myeloid-derived suppressor cells (MDSCs) abundance with differential expression of cPLA2 (PLA2G4A) in invasive breast tumor tissues (source: TISIDB). Microscopic images showing the migrated bone marrow-derived macrophages (BMDM) per field stimulated with CM derived from (C). 231 V and S7OE-231 cells or BMDM preincubated with EP4 receptor antagonist (5 μM L161,982) for 2 h before stimulation with CM of S7OE-231 cells. (D). MVT1 cells treated with VC or 20 μM AACOCF3 (AF3) or BMDM preincubated with EP4 receptor antagonist (5 μM L161,982) for 2 h before stimulation with CM of MVT1 cells. Cell migrations were performed overnight by using transwell migration plates. (E). Flow cytometric analysis of CD206 and MHCII in bone marrow-derived macrophages (BMDM) stimulated with conditioned media (CM) derived from MVT1 cells treated with VC or 20 μM AF3 or BMDM preincubated with EP4 receptor antagonist (5 μM L161,982) for 2 h before stimulation with CM of MVT1 cells.

**Additional file 7: Figure S5**. cPLA2 inhibitor reduced the S100A7-mediated recruitment of tumor-associated macrophages (TAMs) in breast cancer. Flow cytometric analysis of CD11b^+^F4/80^+^ macrophages (out of CD45^+^) in (A). tumors and (B). spleens harvested from MVT1 tumor-bearing mice fed with normal diet (Tet-off) or with doxycycline diet (Tet-on). Bar diagram represents the means ±SEMs of three replicates. (C). t-SNE plots showing the abundance of CD11b^+^F4/80^+^ macrophages in spleens harvested from Tet-off and Tet-on mice treated with VC or AACOCF3 (AF3). (D). KM-plotter survival analysis showed that increased expression of S100A7 mRNA with enriched macrophage correlates with a significantly poor overall survival probability of breast cancer patients (*N* = 547) whereas subjects (*N* = 386) with decreased macrophage infiltration showed insignificant change in survival probability. One way ANOVA was used for multiple group comparisons.

**Additional file 8: Figure S6**. Effect of cPLA2 inhibitor on recruitment of monocytic (M-MDSCs) and polymorphonuclear (PMN-MDSCs) myeloid-derived suppressor cells in syngeneic orthotopic MMTV-rtTA;TetO-mS100a7a15 bi-transgenic mice model. (A). Flow cytometric and (B). t-SNE plot analysis of M-MDSCs (CD11b^+^Gr-1^+^ Ly6C^+^) and PMN-MDSCs (CD11b^+^Gr-1^+^ Ly6G^+^) in spleens harvested from Tet-off and Tet-on mice treated with VC or AF3. Bar diagram represents the means ±SEMs of three replicates. (C). Flow cytometric and (D). t-SNE plot analysis of M-MDSCs and PMN-MDSCs in tumors harvested from Tet-off and Tet-on mice treated with VC or AF3. Bar diagram represents the means ±SEMs of three replicates. ns: non-significant, *P < 0.05, ** *P* < 0.01. One way ANOVA was used for multiple group comparisons.

**Additional file 9: Figure S7**. Supplementary figure related to Fig. 6 (CODEX). Microscopic overlapping multi-color images of FFPE TMAs prepared from of orthotopic syngeneic breast cancer model of mS100a7a15 bitransgenic mouse treated with vehicle control (VC) or cPLA2 inhibitor (AACOCF3). red- CD8a, yellow- CD45R, cyan- CD11b, White- CD90.2/DAPI, blue- CD4, magenta- CD38, and green- Ki67.

**Additional file 10: Figure S8**. Effect of cPLA2 inhibitor on the infiltration of CD4^+^, CD8^+^ tumor-infiltrating lymphocytes (TILs), and abundance of PD-L1^+^ tumor cells in Hu-PDX mouse model. Flow cytometric analysis of (A). CD4^+^ and CD8^+^ TILs (out of EpCAM^−^CD14^−^CD3^+^), (B). PD1^+^ CD4^+^ TILs harvested from Hu-PDX mice treated with 5 mg/kg.bt AACOCF3 (AF3) or vehicle control (VC). Bar diagram represents the means ±SEMs of three replicates. ns: non-significant, *P < 0.05, ** P < 0.01. t test was used for statistical significance.

## Data Availability

All data generated during this study are included in this published article and its supplementary files.

## Abbreviations

cPLA2: Cytosolic phospholipase A2α
PGE2: Prostaglandin E2
iTME: Immunosuppressive tumor microenvironment
TNBC: Triple-negative breast cancer
CODEX: CO-Detection by indEXing
RAGE: Receptor for Advanced Glycation End-products
NSG: NOD scid gamma mouse
MMTV: Mouse mammary tumor virus
VEGF: Vascular endothelial growth factor
TIL: Tumor-infiltrating lymphocytes
PD1: Programmed cell death protein 1
PDL1: Programmed death-ligand 1
CTLA4: Cytotoxic T-Lymphocyte Associated Protein 4
TAM: Tumor-associated macrophages
MDSC: Myeloid-derived suppressor cell
BMDM: Bone marrow-derived macrophages
KM: Kaplan Meier
ROC: Receiver operating characteristics
AUC: Area under the curve
SEEK: Search-Based Exploration of Expression Compendium
TMA: Tissue microarray
HuPDX: Humanized patient-derived xenograft
IHC: Immunohistochemistry
IF: Immunofluorescence
ELISA: Enzyme-linked immunosorbent assay
OSU: Ohio State University
