# Supplementary material for: cPLA2 blockade attenuates S100A7-mediated breast tumorigenicity by inhibiting the immunosuppressive tumor microenvironment
Source: J Exp Clin Cancer Res. 2022 Feb 8;41:54. doi: 10.1186/s13046-021-02221-0 (PMC8822829; doi:10.1186/s13046-021-02221-0)
Supplement: Supplementary file 1 — Additional file 1: Supplementary Table 1: List of reagents and resources. [file 13046_2021_2221_MOESM1_ESM.docx]

**Supplementary Table 1: List of reagents and resources.**

| **Reagents or resources** | **Source** | **Catalog Number** |
| --- | --- | --- |
| ***Antibodies- Flow Cytometry*** |  |  |
| Anti-mouse CD45- Pacific blue | Biolegend | 157212 |
| Anti-mouse CD11b-APC; PerCP | Biolegend | 101230; 101212 |
| Anti-mouse F4/80-PerCP; APC | Biolegend | 123126; 157306 |
| Anti- mouse Gr1-AF594 | Biolegend | 108448 |
| Anti- mouse Ly6C-PE | Biolegend | 128008 |
| Anti- mouse Ly6G-FITC | Biolegend | 127606 |
| Anti- mouse MHCII-AF700 | Biolegend | 107622 |
| Anti- mouse CD206-FITC | Biolegend | 141704 |
| Anti- human CD45-FITC | Biolegend | 982316 |
| Anti- human CD68- A647 | Biolegend | 333820 |
| Anti- human CD163-PerCP/Cy5.5 | Biolegend | 333608 |
| Anti- human CD3-APC/Cy7 | Biolegend | 300318 |
| Anti- human CD4-BV785 | Biolegend | 317442 |
| Anti- human CD8-PE | Biolegend | 303804 |
| Anti- human PD1-PE/Cyanine 7 | Biolegend | 329918 |
| Anti- human PDL1-BV711 | Biolegend | 329722 |
| Anti- human CTLA4-BV605 | Biolegend | 369610 |
| ***Antibody-WB, IHC and IF*** |  |  |
| Anti-mouse S100A7 | Novus Biologicals; Abcam | NB100-56559; ab13680 |
| Anti-mouse RAGE | Santa Cruz | sc-365154 |
| Anti-rabbit S100A7 (mouse) | Abcam | ab275026 |
| Anti-rabbit cPLA2 | Cell Signaling Technology | 5479S |
| Anti-rabbit β-actin | Cell Signaling Technology | [4970](https://www.cellsignal.com/products/primary-antibodies/b-actin-13e5-rabbit-mab/4970?site-search-type=Products&N=4294956287&Ntt=anti-rabbit+%CE%B2-actin&fromPage=plp) |
| Anti-rabbit GAPDH | Cell Signaling Technology | [2118](https://www.cellsignal.com/products/primary-antibodies/gapdh-14c10-rabbit-mab/2118?site-search-type=Products&N=4294956287&Ntt=anti-rabbit+gapdh&fromPage=plp) |
| Donkey anti-rabbit secondary antibody, Alexa Flour 568 | Invitrogen | A10042 |
| Anti-human CD163 | Bio-Rad | ab189915 |
| ***Recombinant proteins and Reagents*** |  |  |
| Human recombinant S100A7 | R&D Systems | 9085-SA-050 |
| Fc receptor blocker | Biolegends | 422302 |
| Fixation/ permeabilization Diluent | Thermoscientific | 00-5223-56 |
| permeabilization buffer | Biolegends | 421002 |
| cPLA2 inhibitor (AACOCF3) | Abcam | ab120350 |
| EP4 inhibitor (L-161,982) | Tocris | 2514 |
| ***Commercial assays*** |  |  |
| S100A7/PGE2 ELISA Kits | Novus Biologicals/R & D | NBP2-31039/KGE004B |
| ImmPACT DAB Substrate Kit, Peroxidase | Vector Laboratories | SK-4100 |

**Abbreviation**: WB, Western Blot; IHC, Immunohistochemistry; IF, Immunofluorescence
