# Supplementary material for: cPLA2 blockade attenuates S100A7-mediated breast tumorigenicity by inhibiting the immunosuppressive tumor microenvironment
Source: J Exp Clin Cancer Res. 2022 Feb 8;41:54. doi: 10.1186/s13046-021-02221-0 (PMC8822829; doi:10.1186/s13046-021-02221-0)
Supplement: Supplementary file 2 — Additional file 2: Supplementary Table 2: Clinicopathological detail of Tissue microarray (BR1002b). [file 13046_2021_2221_MOESM2_ESM.docx]

**Supplementary Table 2: Clinicopathological detail of Tissue microarray (BR1002b)**

| **Age** | **Pathology diagnosis** | **TNM** | **Grade** | **Stage** | **Type** | **Tissue ID.** |
| --- | --- | --- | --- | --- | --- | --- |
| 81 | Invasive ductal carcinoma | T3N0M0 | 1 | IIB | Malignant | Fmg020310 |
| 45 | Invasive ductal carcinoma | T4N2M0 | 1 | IIIB | Malignant | Fmg010767 |
| 47 | Invasive ductal carcinoma | T2N0M0 | 1 | IIA | Malignant | Fmg010756 |
| 30 | Invasive ductal carcinoma | T2N0M0 | 1 | IIA | Malignant | Fmg020232 |
| 64 | Invasive ductal carcinoma | T2N0M0 | 1 | IIA | Malignant | Fmg010825 |
| 47 | Invasive ductal carcinoma | T2N0M0 | 1 | IIA | Malignant | Fmg010778 |
| 45 | Invasive ductal carcinoma | T2N0M0 | 1 | IIA | Malignant | Fmg010942 |
| 54 | Invasive ductal carcinoma | T2N0M0 | 1 | IIA | Malignant | Fmg010637 |
| 48 | Invasive ductal carcinoma | T2N0M0 | 1 | IIA | Malignant | Fmg020244 |
| 52 | Invasive ductal carcinoma | T2NIM0 | 1 | IIB | Malignant | Fmg020537 |
| 47 | Invasive ductal carcinoma | T2N0M0 | 1 | IIA | Malignant | Fmg020984 |
| 40 | Invasive ductal carcinoma | T2N0M0 | 1 | IIA | Malignant | Fmg020761 |
| 57 | Invasive ductal carcinoma | T2N0M0 | 1 | IIA | Malignant | Fmg020558 |
| 65 | Invasive ductal carcinoma | T2N0M0 | 1 | IIA | Malignant | Fmg020548 |
| 38 | Invasive ductal carcinoma | T2NIM0 | 1 | IIB | Malignant | Fmg010593 |
| 68 | Invasive ductal carcinoma | T4bN0M0 | 2 | IIIB | Malignant | Fmg010927 |
| 46 | Invasive ductal carcinoma | T2N0M0 | 2 | IIA | Malignant | Fmg020029 |
| 60 | Invasive ductal carcinoma | T2N0M0 | 2 | IIA | Malignant | Fmg020754 |
| 60 | Invasive ductal carcinoma | T4N0M0 | 2 | IIIB | Malignant | Fmg040174 |
| 47 | Invasive ductal carcinoma | T2N0M0 | 2 | IIA | Malignant | Fmg010761 |
| 37 | Invasive ductal carcinoma | T2NIM0 | 2 | IIB | Malignant | Fmg010612 |
| 38 | Invasive ductal carcinoma | T2NIM0 | 2 | IIB | Malignant | Fmg010758 |
| 59 | Invasive ductal carcinoma | T2N0M0 | 2 | IIA | Malignant | Fmg010639 |
| 45 | Invasive ductal carcinoma | T2N0M0 | 2 | IIA | Malignant | Fmg010622 |
| 48 | Invasive ductal carcinoma | T4N2M0 | 2 | IIIB | Malignant | Fmg010531 |
| 79 | Invasive ductal carcinoma | T2NIM0 | 2 | IIB | Malignant | Fmg010491 |
| 54 | Invasive ductal carcinoma | T2NIM0 | 2 | IIB | Malignant | Fmg010396 |
| 71 | Invasive ductal carcinoma | T2N0M0 | 2 | IIA | Malignant | 064809C2 |
| 65 | Invasive ductal carcinoma | T2NIM0 | 3 | IIB | Malignant | Fmg020641 |
| 35 | Invasive ductal carcinoma | T3N0M0 | 3 | IIB | Malignant | Fmg020464 |
| 63 | Invasive ductal carcinoma | T2N0M0 | 3 | IIA | Malignant | Fmg050622 |
| 57 | Invasive ductal carcinoma | T2N0M0 | 3 | IIA | Malignant | Fmg020341 |
| 53 | Invasive ductal carcinoma | T2N0M0 | 3 | IIA | Malignant | Fmg010928 |
| 62 | Invasive ductal carcinoma | T2N0M0 | 3 | IIA | Malignant | Fmg050602 |
| 40 | Invasive ductal carcinoma | T2N0M0 | 3 | IIA | Malignant | Fmg040783 |
| 49 | Invasive ductal carcinoma | T2N0M0 | 3 | IIA | Malignant | Fmg020252 |
| 60 | Normal breast tissue | - | - | - | Normal | Fmg010510 |
| 40 | Normal breast tissue | - | - | - | Normal | Fmg021239 |
| 51 | Normal breast tissue | - | - | - | Normal | Fmg032245 |
| 51 | Normal breast tissue | - | - | - | Normal | Fmg010306 |
| 38 | Normal breast tissue | - | - | - | Normal | Fmg020143 |
| 44 | Normal breast tissue | - | - | - | Normal | Fmg010324 |
| 47 | Normal breast tissue | - | - | - | Normal | Fmg010230 |
| 44 | Normal breast tissue | - | - | - | Normal | Fmg010542 |
| 46 | Normal breast tissue | - | - | - | Normal | Fmg010594 |
| 40 | Normal breast tissue | - | - | - | Normal | Fmg100228 |
| 41 | Normal breast tissue | - | - | - | Normal | Fmg021705 |
| 45 | Normal breast tissue | - | - | - | Normal | Fmg020565 |
| 39 | Normal breast tissue | - | - | - | Normal | Fmg010611 |
| 57 | Normal breast tissue | - | - | - | Normal | Fmg020933 |
| 42 | Normal breast tissue | - | - | - | Normal | Fmg020849 |
| 43 | Normal breast tissue | - | - | - | Normal | Fmg020666 |
| 37 | Normal breast tissue | - | - | - | Normal | Fmg020550 |
| 35 | Normal breast tissue | - | - | - | Normal | Fmg010800 |
| 44 | Normal breast tissue | - | - | - | Normal | Fmg100252 |
| 41 | Normal breast tissue | - | - | - | Normal | Fmg100242 |
| 53 | Normal breast tissue | - | - | - | Normal | 198693 |
| 50 | Normal breast tissue | - | - | - | Normal | Fmg010321 |
| 42 | Normal breast tissue | - | - | - | Normal | Fmg010940 |
| 53 | Normal breast tissue | - | - | - | Normal | 170850A1 |
| 39 | Normal breast tissue | - | - | - | Normal | 174747A3 |
| 44 | Normal breast tissue | - | - | - | Normal | Fmg021291 |
| 38 | Normal breast tissue | - | - | - | Normal | Fmg021401 |
| 50 | Normal breast tissue | - | - | - | Normal | Fmg031420 |
| 55 | Normal breast tissue | - | - | - | Normal | 199428 |
| 41 | Normal breast tissue | - | - | - | Normal | Fmg010938 |
| 42 | Normal breast tissue | - | - | - | Normal | Fmg010609 |
| 85 | Normal breast tissue | - | - | - | Normal | 200371 |
| 54 | Normal breast tissue | - | - | - | Normal | 200063 |
| 49 | Normal breast tissue | - | - | - | Normal | Fmg020196 |
| 43 | Normal breast tissue | - | - | - | Normal | 199640 |
| 44 | Normal breast tissue | - | - | - | Normal | Fmg010621 |
| 42 | Normal breast tissue | - | - | - | Normal | Fmg010188 |
| 76 | Normal breast tissue | - | - | - | Normal | 197963 |
| 45 | Normal breast tissue | - | - | - | Normal | Fmg010231 |
| 53 | Normal breast tissue | - | - | - | Normal | Fmg010272 |
| 42 | Normal breast tissue | - | - | - | Normal | 198985 |
| 48 | Normal breast tissue | - | - | - | Normal | 199840 |
| 27 | Normal breast tissue | - | - | - | Normal | Fmg07N025 |
| 35 | Normal breast tissue | - | - | - | Normal | Fmg07N011 |
| 21 | Normal breast tissue | - | - | - | Normal | Fmg06N024 |
| 19 | Normal breast tissue | - | - | - | Normal | Fmg07N013 |
