# Supplementary figures and images for: cPLA2 blockade attenuates S100A7-mediated breast tumorigenicity by inhibiting the immunosuppressive tumor microenvironment
Source: J Exp Clin Cancer Res. 2022 Feb 8;41:54. doi: 10.1186/s13046-021-02221-0 (PMC8822829; doi:10.1186/s13046-021-02221-0)

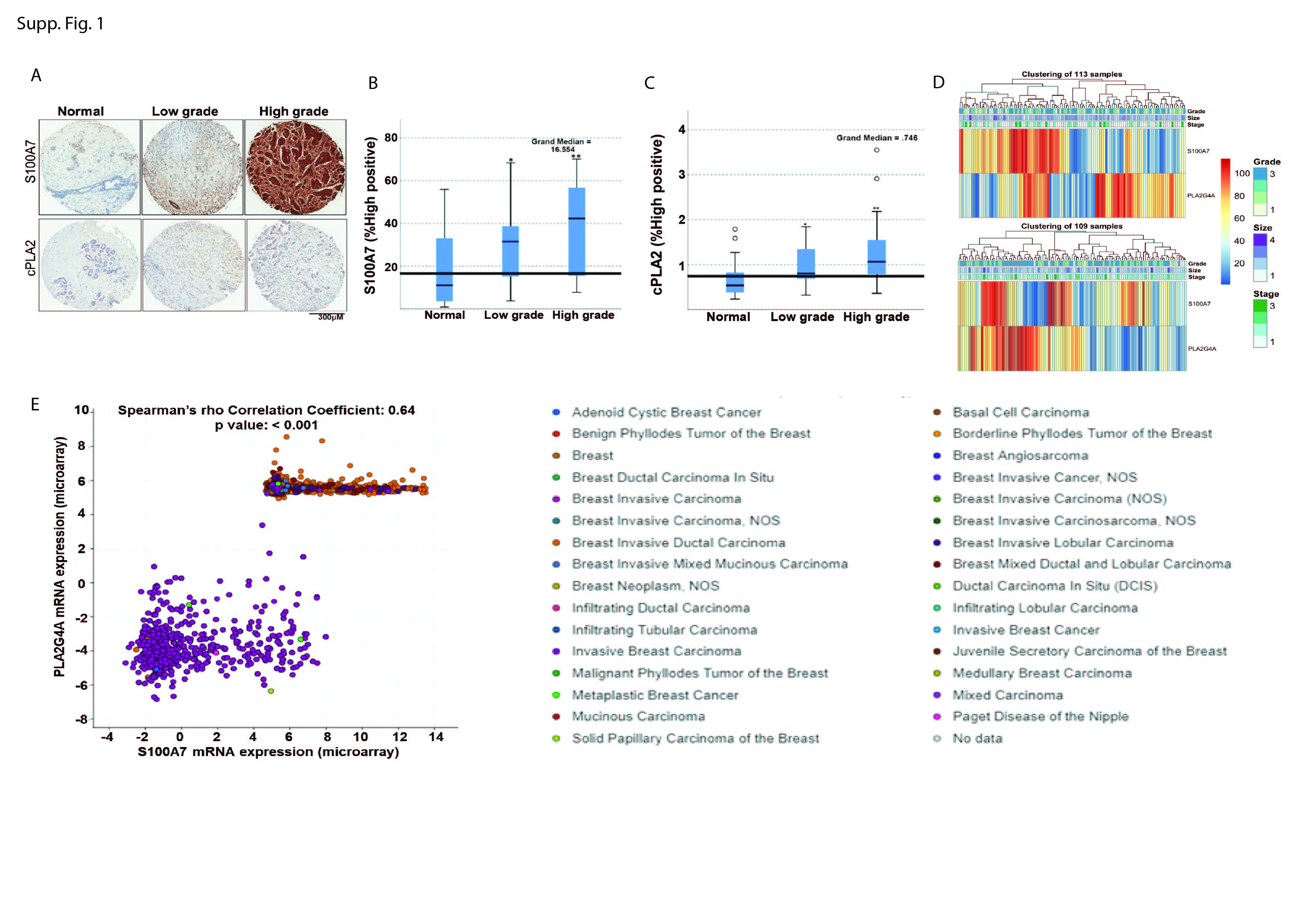

Supplement: Supplementary file 3 — Additional file 3: Figure S1. Expression and correlation of S100A7 and cPLA2 in breast cancer types. (A). Representative S100A7 and cPLA2 immunohistochemistry (IHC) images of invasive breast cancer specimens. [source: US Biomax]. Box plot showing percent (%) high positive (B). S100A7 and (C). cPLA2 stained cells in normal (n = 46), low-grade (n = 15), and high-grade (n = 21) breast cancer specimens. Non-parametric test (Independent-Samples Median Test) was used to calculate p values. (D). Heat map analysis showing the differential expression of S100A7 and PLA2G4A across different grades, size, and stages of breast cancer patients using Caldas (n = 113) & Chin; (n = 109) datasets. (E). Correlation analysis of S100A7 and PLA2G4A was analyzed in different breast cancer types (n = 3380) using cBioPortal for Cancer Genomics. *P < 0.05*, *P < 0.01, ***P < 0.001. [file 13046_2021_2221_MOESM3_ESM.jpg]

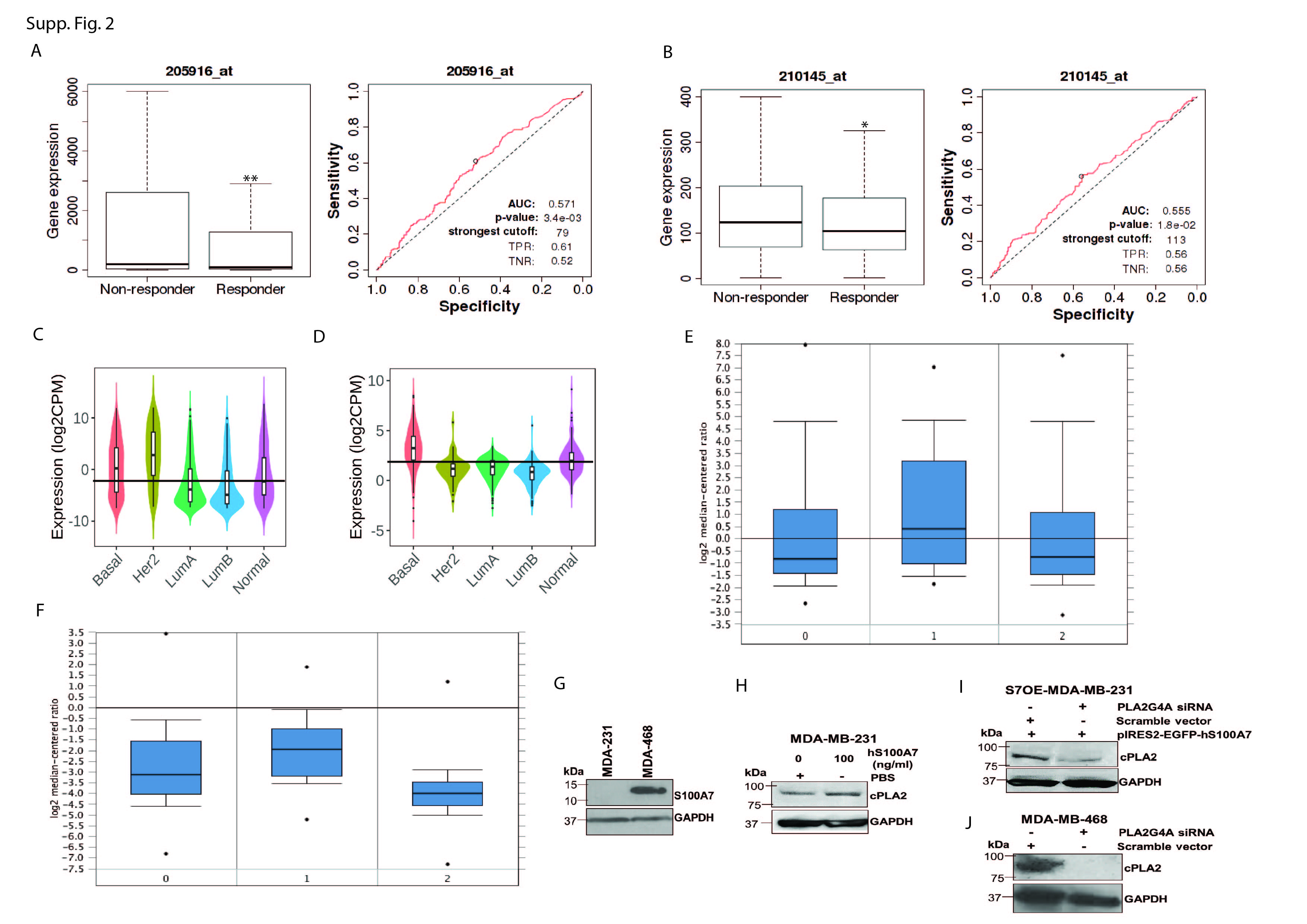

Supplement: Supplementary file 4 — Additional file 4: Figure S2. Effect of S100A7/cPLA2 gene expression on recurrence free survival of breast cancer patients. (A). ROC plotter analysis of S100A7 (205916_at; Mann-Whitney test p-value: 0.0076) and (B). cPLA2 (210145_at; Mann-Whitney test p-value: 0.037) for relapse free survival at 5 years after treatment with any chemotherapy in breast cancer patients. The graphs were plotted by using ROC plotter online database (http://www.rocplot.org/) with responder (n = 256) and non-responder (n = 220) breast cancer patients. Gene expression of (C). S100A7 and (D). PLA2G4A were analyzed in normal subjects (n = 137) and different breast cancer subtypes (Basal = 172; Her2 = 73, Luminal A = 508 and Luminal B = 191) using TISIDB database. (E). S100A7 (Reporter: A_23_P103310) and (F). PLA2G4A (Reporter: A_23_P11682) mRNA expressions were analyzed in triple negative (TNBC) and other biomarker status of invasive ductal breast carcinoma using TCGA breast cancer dataset of Oncomine database. [0 = no value or unidentified hormonal status (n = 297), 1 = HER2/ER/PR negative or TNBC (n = 46), 2 = other breast cancer biomarkers (n = 250)]. (G). Immunoblot analysis of S100A7 in MDA-MB-231 and MDA-MB-468 cells. (H). Effect of recombinant human S100A7 treatment (100 ng/ml for 24 h) on cPLA2 expression in MDA-MB-231 cells. Immunoblot analysis of cPLA2 in (I). S7OE-231 and (J). MDA-MB-468 cells transiently transfected with control or cPLA2-siRNA. [file 13046_2021_2221_MOESM4_ESM.jpg]

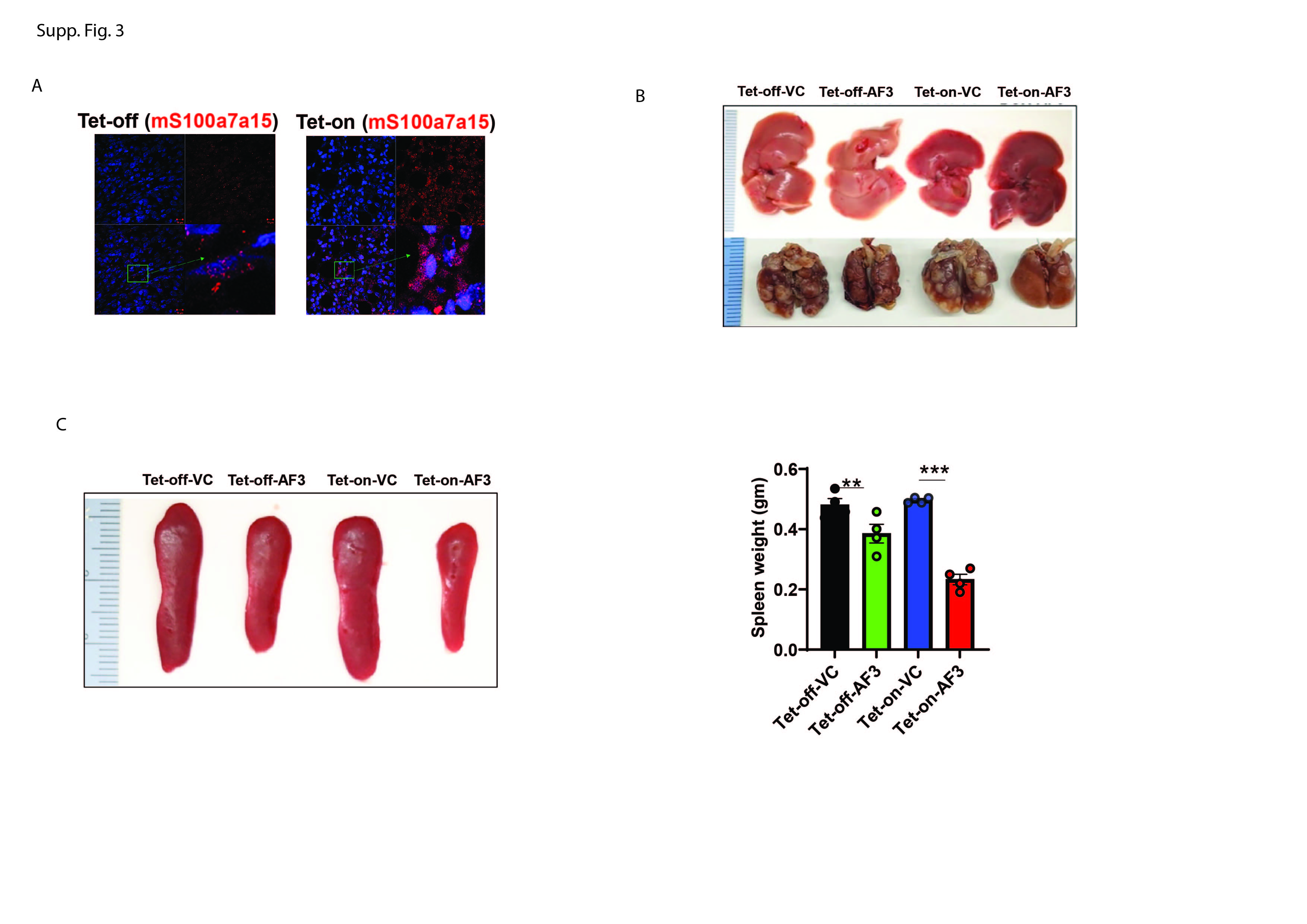

Supplement: Supplementary file 5 — Additional file 5: Figure S3. Pharmacological inhibition of cPLA2 suppress the S100A7-mediated metastasis and gain in spleen weight in syngeneic orthotopic MMTV-rtTA;TetO-mS100a7a15 bi-transgenic mice model. (A). Immunofluorescence (IF) analysis of mS100a7a15 expression in tumor tissues harvested from MVT1 tumor-bearing mice fed with normal diet (Tet-off) or with doxycycline diet (Tet-on). (B). Representative images of livers (top) and lungs (bottom) harvested from Tet-off and Tet-on mice treated with VC or AF3. (C). Representative images of spleens harvested from Tet-off and Tet-on mice treated with vehicle control (VC) or 5 mg/kg.bt of AACOCF3 (AF3). Bar diagram represents the means of spleen weight (gm) ± SEMs of four replicates. * P < 0.05, *** P < 0.001. One way ANOVA was used for multiple group comparisons. [file 13046_2021_2221_MOESM5_ESM.jpg]

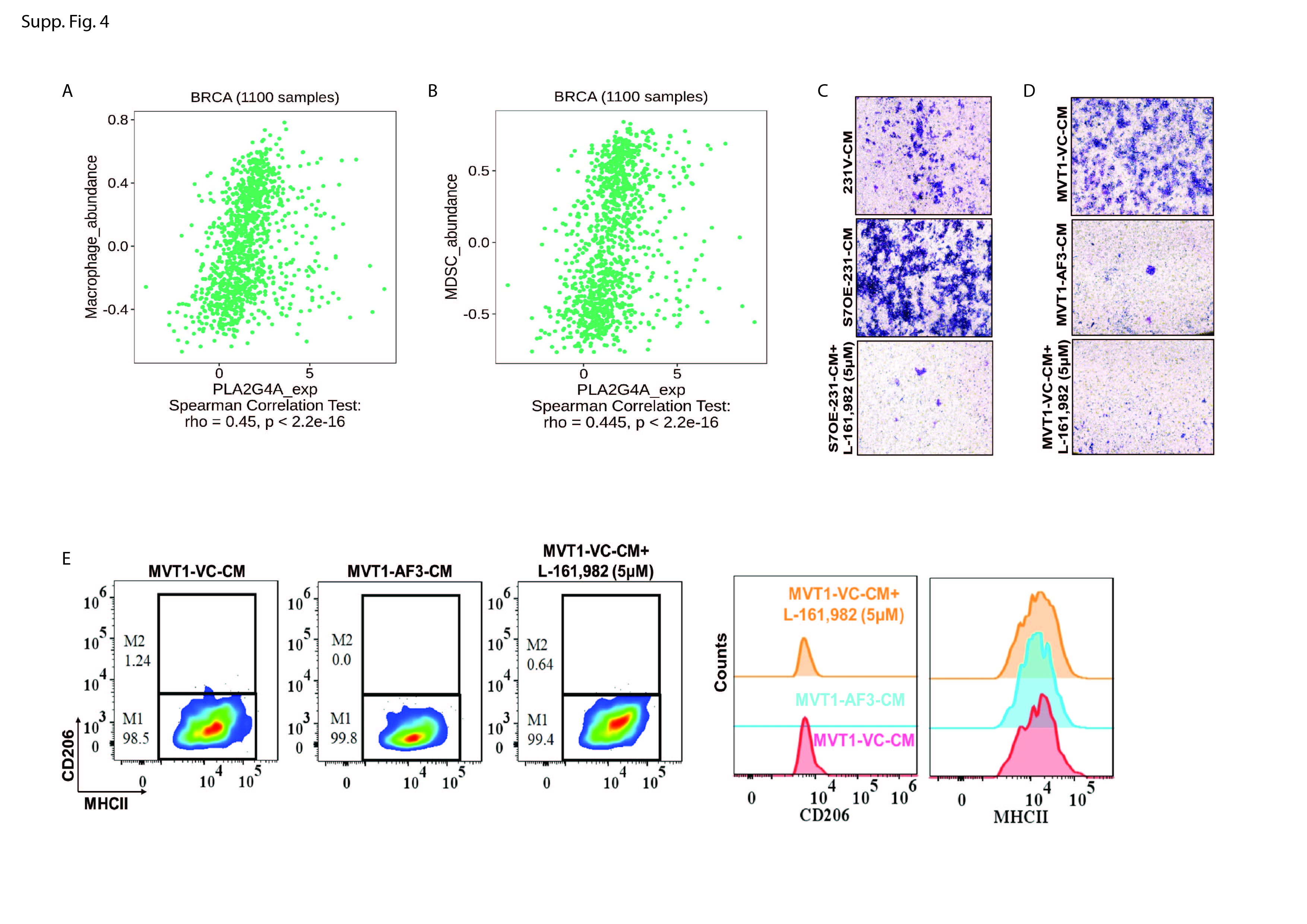

Supplement: Supplementary file 6 — Additional file 6: Figure S4. S100A7/cPLA2 signaling regulates the recruitment and plasticity of myeloid cells in breast cancer. Correlation analysis of (A). Macrophage and (B). Myeloid-derived suppressor cells (MDSCs) abundance with differential expression of cPLA2 (PLA2G4A) in invasive breast tumor tissues (source: TISIDB). Microscopic images showing the migrated bone marrow-derived macrophages (BMDM) per field stimulated with CM derived from (C). 231 V and S7OE-231 cells or BMDM preincubated with EP4 receptor antagonist (5 μM L161,982) for 2 h before stimulation with CM of S7OE-231 cells. (D). MVT1 cells treated with VC or 20 μM AACOCF3 (AF3) or BMDM preincubated with EP4 receptor antagonist (5 μM L161,982) for 2 h before stimulation with CM of MVT1 cells. Cell migrations were performed overnight by using transwell migration plates. (E). Flow cytometric analysis of CD206 and MHCII in bone marrow-derived macrophages (BMDM) stimulated with conditioned media (CM) derived from MVT1 cells treated with VC or 20 μM AF3 or BMDM preincubated with EP4 receptor antagonist (5 μM L161,982) for 2 h before stimulation with CM of MVT1 cells. [file 13046_2021_2221_MOESM6_ESM.jpg]

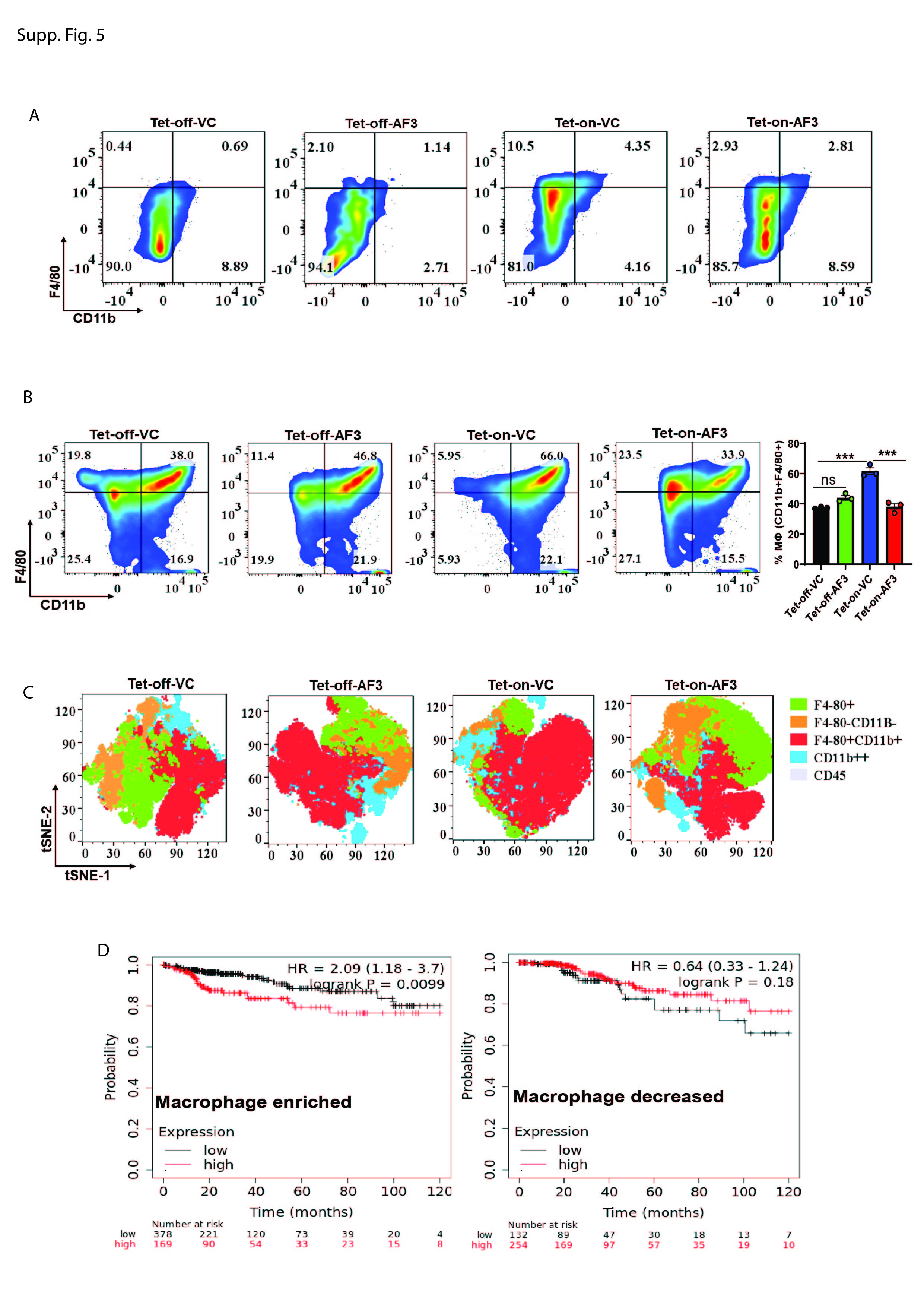

Supplement: Supplementary file 7 — Additional file 7: Figure S5. cPLA2 inhibitor reduced the S100A7-mediated recruitment of tumor-associated macrophages (TAMs) in breast cancer. Flow cytometric analysis of CD11b+F4/80+ macrophages (out of CD45+) in (A). tumors and (B). spleens harvested from MVT1 tumor-bearing mice fed with normal diet (Tet-off) or with doxycycline diet (Tet-on). Bar diagram represents the means ±SEMs of three replicates. (C). t-SNE plots showing the abundance of CD11b+F4/80+ macrophages in spleens harvested from Tet-off and Tet-on mice treated with VC or AACOCF3 (AF3). (D). KM-plotter survival analysis showed that increased expression of S100A7 mRNA with enriched macrophage correlates with a significantly poor overall survival probability of breast cancer patients (N = 547) whereas subjects (N = 386) with decreased macrophage infiltration showed insignificant change in survival probability. One way ANOVA was used for multiple group comparisons. [file 13046_2021_2221_MOESM7_ESM.jpg]

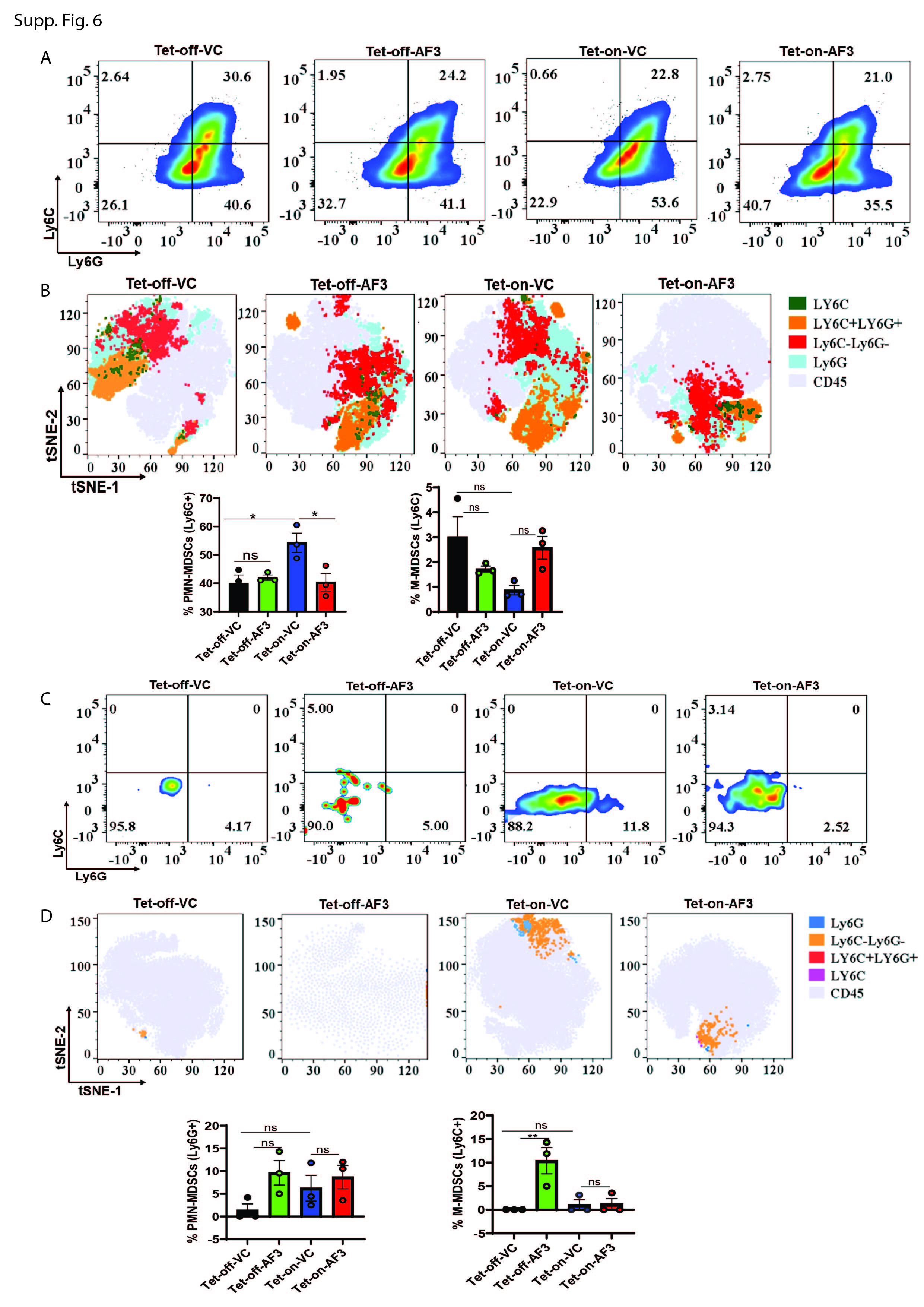

Supplement: Supplementary file 8 — Additional file 8: Figure S6. Effect of cPLA2 inhibitor on recruitment of monocytic (M-MDSCs) and polymorphonuclear (PMN-MDSCs) myeloid-derived suppressor cells in syngeneic orthotopic MMTV-rtTA;TetO-mS100a7a15 bi-transgenic mice model. (A). Flow cytometric and (B). t-SNE plot analysis of M-MDSCs (CD11b+Gr-1+ Ly6C+) and PMN-MDSCs (CD11b+Gr-1+ Ly6G+) in spleens harvested from Tet-off and Tet-on mice treated with VC or AF3. Bar diagram represents the means ±SEMs of three replicates. (C). Flow cytometric and (D). t-SNE plot analysis of M-MDSCs and PMN-MDSCs in tumors harvested from Tet-off and Tet-on mice treated with VC or AF3. Bar diagram represents the means ±SEMs of three replicates. ns: non-significant, *P < 0.05, ** P < 0.01. One way ANOVA was used for multiple group comparisons. [file 13046_2021_2221_MOESM8_ESM.jpg]

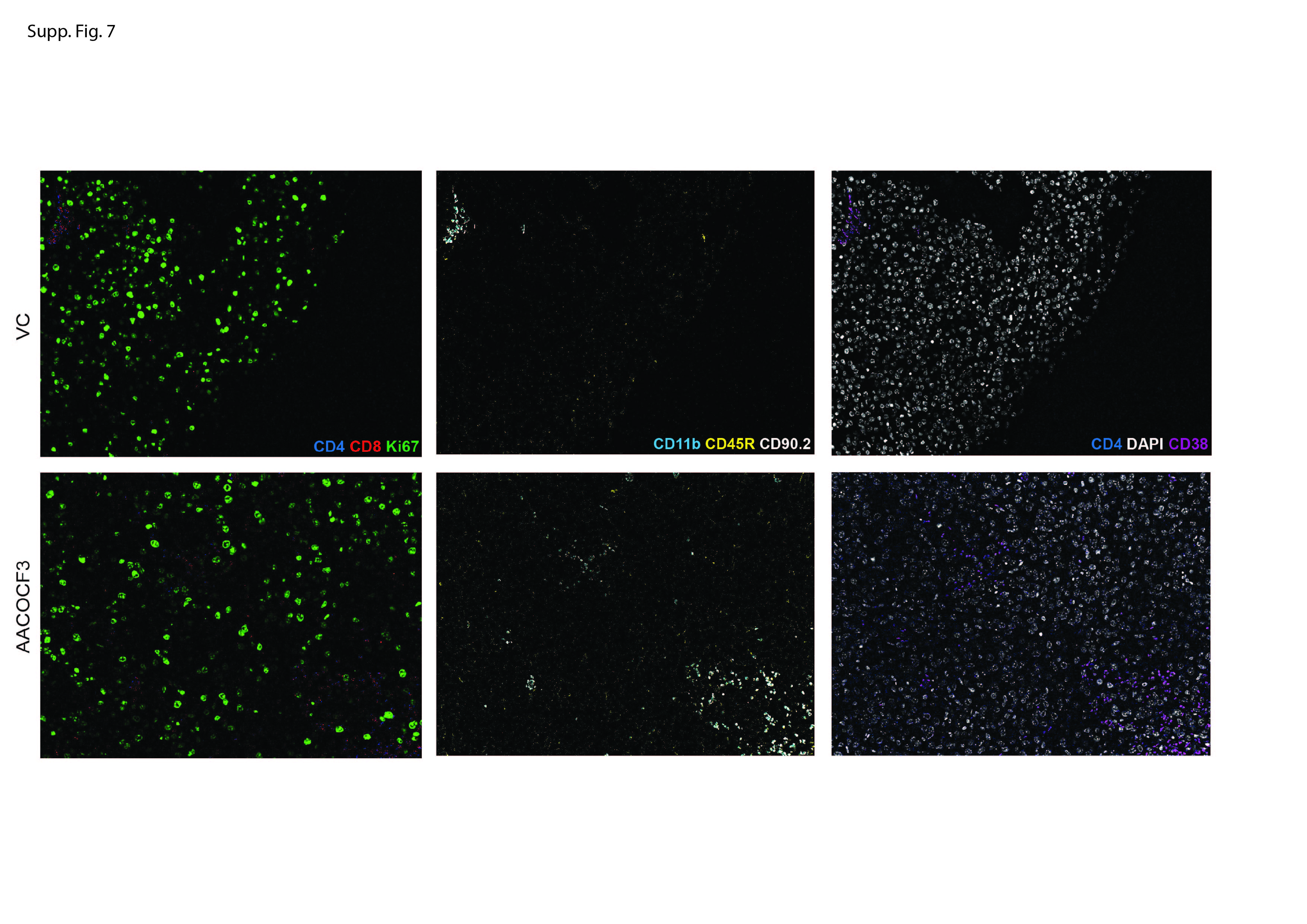

Supplement: Supplementary file 9 — Additional file 9: Figure S7. Supplementary figure related to Fig. 6 (CODEX). Microscopic overlapping multi-color images of FFPE TMAs prepared from of orthotopic syngeneic breast cancer model of mS100a7a15 bitransgenic mouse treated with vehicle control (VC) or cPLA2 inhibitor (AACOCF3). red- CD8a, yellow- CD45R, cyan- CD11b, White- CD90.2/DAPI, blue- CD4, magenta- CD38, and green- Ki67. [file 13046_2021_2221_MOESM9_ESM.jpg]

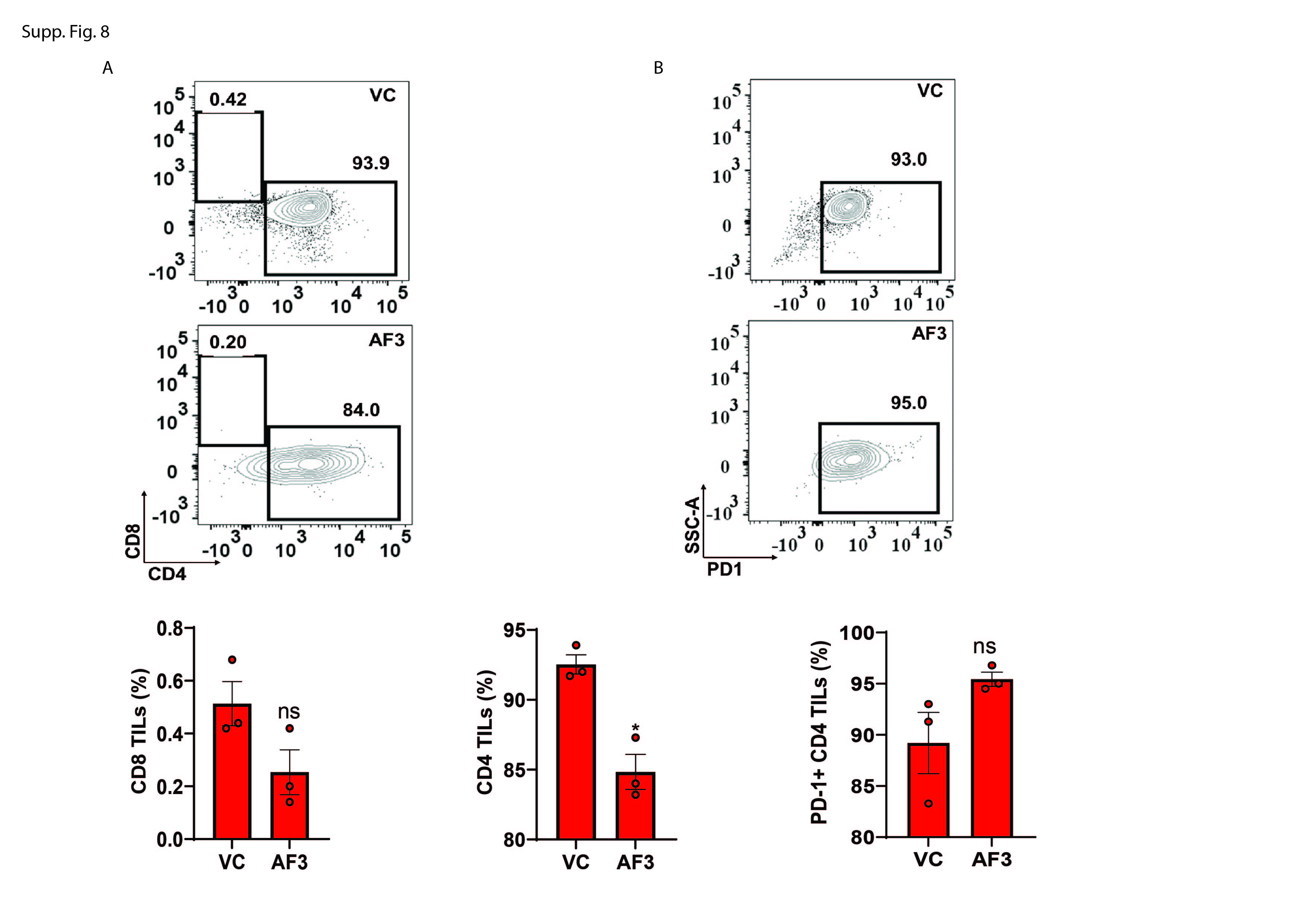

Supplement: Supplementary file 10 — Additional file 10: Figure S8. Effect of cPLA2 inhibitor on the infiltration of CD4+, CD8+ tumor-infiltrating lymphocytes (TILs), and abundance of PD-L1+ tumor cells in Hu-PDX mouse model. Flow cytometric analysis of (A). CD4+ and CD8+ TILs (out of EpCAM−CD14−CD3+), (B). PD1+ CD4+ TILs harvested from Hu-PDX mice treated with 5 mg/kg.bt AACOCF3 (AF3) or vehicle control (VC). Bar diagram represents the means ±SEMs of three replicates. ns: non-significant, *P < 0.05, ** P < 0.01. t test was used for statistical significance. [file 13046_2021_2221_MOESM10_ESM.jpg]
